# Quantum-Electrodynamical Density-Functional Theory Exemplified by the Quantum Rabi Model

**DOI:** 10.1021/acs.jpca.4c07690

**Published:** 2025-02-19

**Authors:** Vebjørn H. Bakkestuen, Vegard Falmår, Maryam Lotfigolian, Markus Penz, Michael Ruggenthaler, Andre Laestadius

**Affiliations:** † Department of Computer Science, Oslo Metropolitan University, Oslo 0130, Norway; ‡ Max Planck Institute for the Structure and Dynamics of Matter and Center for Free-Electron Laser Science, Hamburg 22761, Germany; § The Hamburg Center for Ultrafast Imaging, Hamburg 22761, Germany; ∥ Hylleraas Centre for Quantum Molecular Sciences, Department of Chemistry, University of Oslo, Oslo 0315, Norway

## Abstract

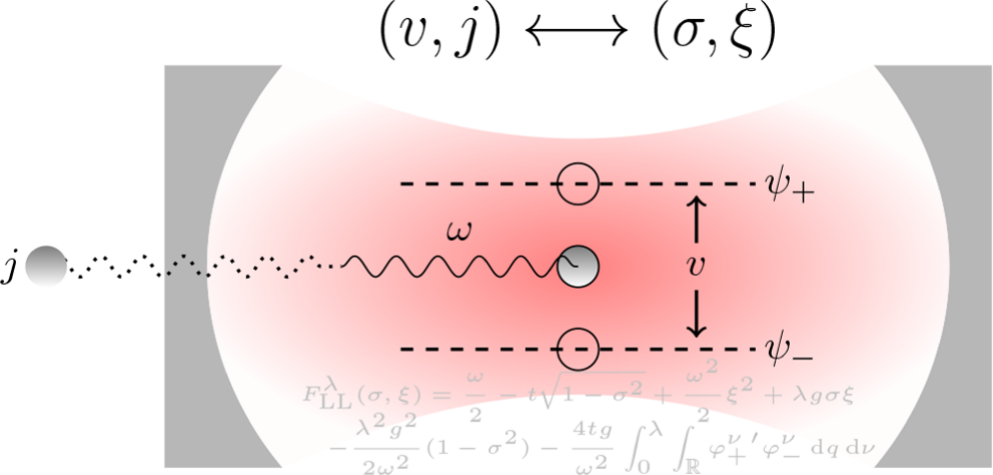

The key features of density-functional theory (DFT) within a minimalistic implementation of quantum electrodynamics are demonstrated, thus allowing to study elementary properties of quantum-electrodynamical density-functional theory (QEDFT). We primarily employ the quantum Rabi model that describes a two-level system coupled to a single photon mode and also discuss the Dicke model, where multiple two-level systems couple to the same photon mode. In these settings, the density variables of the system are the polarization and the displacement of the photon field. We give analytical expressions for the constrained-search functional and the exchange-correlation potential and compare them to established results from QEDFT. We further derive a form for the adiabatic connection that is almost explicit in the density variables, up to only a nonexplicit correlation term that gets bounded both analytically and numerically. This allows several key features of DFT to be studied without approximations.

## 1 Introduction

### 1.1 Prelude and Overview

The study of light–matter interactions forms the basis for understanding a wide range of phenomena whose effects are instrumental for measuring and manipulating matter in experiments. At the fundamental level, charged particles interact among each other through their coupling to the photon field, a process that is described by quantum electrodynamics (QED).^1−6^ While the quantization of the electromagnetic field is often considered to only be relevant for high-energy physics, QED effects, such as spontaneous emission or the Purcell effect, also occur in the low-energy (nonrelativistic) regime of charged particles. In recent years, many experimental and theoretical works have shown that in optical environments, such as Fabry–Pérot cavities, changes in the quantized light field can modify chemical and material properties even at equilibrium.^7−9^ It has therefore become increasingly relevant to extend well-established first-principles methods, such as density-functional theory (DFT) and coupled-cluster theory, to encompass QED.^10−14^

Due to its computational simplicity, DFT is the method predominantly used when studying quantum systems with large numbers of particles. In DFT, the *N*-body wave function—with its intractable dimensionality—is replaced by the one-body particle density. This dimensional reduction is precisely why DFT calculations provide effective approximations and thus have become an indispensable tool across many fields, such as chemistry, materials science and solid-state physics.^15−17^ Since the seminal papers of Hohenberg and Kohn,^18^ Kohn and Sham,^19^ and Lieb,^20^ significant efforts have been devoted both to the numerical and mathematical developments of DFT. Besides, motivated by its extremely elegant formulation in terms of convex analysis, a perspective toward DFT has emerged that makes it more than just an approximation method. The concave form of the ground-state energy in terms of the external potential naturally yields the universal functional as its Legendre–Fenchel transform. Then the Hohenberg–Kohn theorem is the statement that the subdifferential of the universal functional just contains a single element (see also Section 1.4) and *v*-representability connects closely to differentiability of this functional. The whole of DFT thus follows as a convex treatment of many-body quantum mechanics in the ground state. In this sense, DFT can be referred to more as a discovery than an invention.^21^ The DFT formulation of QED presented here serves a case in point for this viewpoint.

In this article we will exemplify several key features of DFT by derivations and examples using simple models for QED—settings that allow significantly more explicit constructions than the standard Coulombic DFT. This means the whole formalism of DFT can be well-defined and important results like the Hohenberg–Kohn theorem or *v*-representability receive a mathematical rigorous treatment. Apart from formulating the basics of how to build up QEDFT, this has the additional value of constituting a pedagogical showcase for DFT in general. The richness of mathematical and physical concepts that enter the theory will become clearly appreciable in the course of this work. In this sense, this complements our previous, more technical work that aimed at defining the basic DFT structures for the Dicke model.^22^

The article is structured as follows. This introductory section continues with a short overview of quantum-electrodynamical density-functional theory (QEDFT) and briefly explain the landscape and hierarchy of QED models surrounding our model of choice, the *quantum Rabi model*. We conclude the section with a summary of important concepts in the mathematical formulation of standard DFT. The quantum Rabi model setting, including the Hamiltonian, general ground-state properties, and hyper-virial results are presented in Section 2. Note that Sections 2.5 and 3.3 pertain to a generalization to the Dicke model. In a first reading these sections may be safely skipped as they do not directly affect the analysis of the quantum Rabi model, however they illustrate important aspects of DFT not readily seen in the simpler setting. Section 3 introduces the polarization and displacement as internal variables of this QEDFT formulation and establishes the corresponding Hohenberg–Kohn-type results. The DFT theory part is then continued by defining the Levy–Lieb constrained-search functional and studying its properties for the model at hand in Section 4. This also establishes the important result that all internal-variable pairs are in fact *v*-representable if they are not on the boundary of their domain. As an important tool of DFT the adiabatic connection is analyzed in Section 5, where we can give an almost explicit form due to the relative simplicity of the model. Finally, functional approximation that are based on a photon-free formulation are discussed in Section 6 and we compare to other results from QEDFT. We conclude in Section 7.

### 1.2 Quantum-Electrodynamical Density-Functional Theory

Of specific interest for this work is the extension of DFT to QED, which has been termed QEDFT and has been investigated for the static^11,23^ as well as the time-dependent (and even relativistic) case.^10,24−28^ As a formulation, QEDFT has been applied to a broad variety of physical and chemical situations and different approximations to the new photon-matter exchange-correlation field in the Kohn–Sham formulation of QEDFT have been proven to provide accurate results.^29−35^ Yet a detailed mathematical investigation of this new form of a density-functional reformulation, similar to how it was performed in standard DFT,^20,36,37^ has so far not been pursued, apart from Bakkestuen et al.^22^ that now led to this work. Such an investigation is, however, important not only as fundamental question, but also to further guide the development of QEDFT and its approximation strategies.

While most of the rigorous considerations in QEDFT are based on the Pauli–Fierz Hamiltonian,^6^ various approximations to this Hamiltonian are used as a starting point for further investigations.^9^ These approximate Hamiltonians lead to a hierarchy of QEDFTs^10,27^ and yield a connection to well-established models of quantum optics that are designed to describe the photonic subsystem well, while significantly simplifying the matter part. One such paradigmatic quantum-optical model is the quantum Rabi model,^38^ which will be the main focus of this article. Given the vast number of models available, let us briefly orient ourselves on where in the landscape of QED models our investigation will take place, before turning to the analysis.

### 1.3 Models in QED

QED is arguably the most accurate description of light-matter interactions and as a field it is as old as quantum mechanics itself.^1^ It is described by the QED Lagrangian , and its equations of motion are the relativistic Dirac equation and the wave equation for the vector potential.^1−3^ Although QED is one of the most accurate descriptions of nature ever conceived and has been studied for almost 100 years, it is full of challenges. Notably, within the full field-theoretic treatment QED processes are calculable only perturbatively and the complexity of the calculations rapidly increases in the perturbative expansion. Moreover, most applications in chemistry and solid-state physics involve energies that are not sufficiently large that relativistic effects become important, at least not for the fermionic degrees of freedom. In these applications, the treatment of the fully relativistic Dirac equation thus becomes superfluous. Within low-energy applications of QED one usually chooses the Coulomb gauge, also known as the radiation or transversal gauge, as it has a number of useful properties, especially in the semiclassical regime (quantum particles and classical radiation fields). In particular it removes the unphysical degrees of freedom in the gauge field, ensuring that the vector potential only has the two transversal polarizations, and it picks out the Coulomb potential to describe interactions between the fermions.

**Figure 1:**
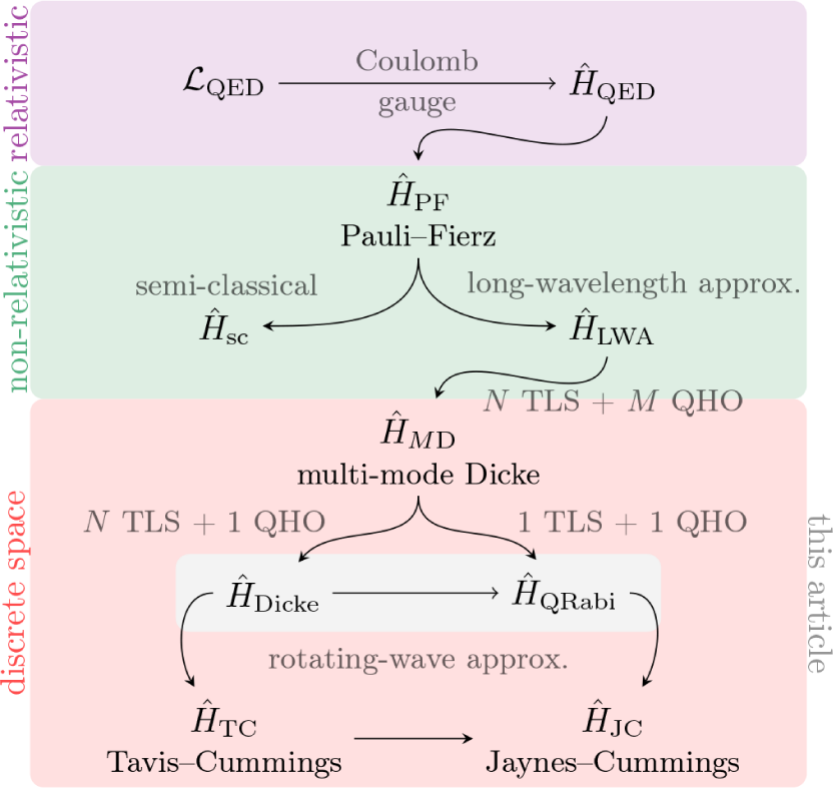
Hierarchy of QED models relevant for the current analysis. The top panel (violet) contains the fully relativistic theory, the middle panel (green) is the nonrelativistic limit in form of the Pauli–Fierz Hamiltonian and further simplifications, and bottom panel (red) has different models following from discretizations. The models of interest in this article are marked by the injected gray panel, of particular interest is the quantum Rabi model.

The wide range of possible simplifications of the theory gives rise to a large hierarchy of models. A small snapshot of such models is outlined in Figure 1. Let us briefly comment on how they connect to each other. Starting from , there are a multitude of gauge-fixing conditions, each with a particular set of advantages and disadvantages. By choosing the Coulomb gauge and equal-time commutation relations, one arrives at what we will refer to as the *relativistic QED HamiltonianĤ*_QED_ (as the volume integral over the QED Hamiltonian density). Then, in taking the nonrelativistic (low energy) limit, one arrives at the *Pauli–Fierz HamiltonianĤ*_PF_,^6^ where usually the Born–Oppenheimer approximation is already included. A further important simplification is the long-wavelength or dipole approximation, where the transfer of momentum between light and matter is assumed to be zero.^9^ In this context, we note that different forms of the resulting Hamiltonian are possible. In certain forms besides the Coulomb matter–matter interaction, photon-induced direct matter–matter coupling terms appear, which are called dipole-self-energies or self-polarization terms.^39,40^ In the following we discard such direct matter–matter interaction terms that arise due to further transformations of the dipole-approximated Pauli–Fierz Hamiltonian but want to highlight that these terms can become important in certain situations.^41^ Despite simplifications, such models are still difficult to solve. Thus, many applications rely on further approximations and one possible avenue is to describe the light part classically while keeping the matter part quantum, which yields what is usually referred to as a *semiclassical* approach. An example of such approach is the WKB (Wentzel–Kramers–Brillouin) approximation. On the other side, the models we are interested in here use discretization in both the light and matter parts, but retain the “quantumness” of both. For such models, one picks out *M* photonic modes from the expansion of the vector-potential operator and discards the rest, an efficient approach when only a limited number of photonic modes actively couple to the problem. This allows the photonic sector to be treated as *M* quantum harmonic oscillators (QHO). If one further simplifies the setting by treating the fermionic sector as *N* two-level systems (TLS), one obtains what we refer to as the *multimode Dicke HamiltonianĤ*_MD_.

Of particular interest for this article are two variants of the multimode Dicke model. In particular, the restriction to just one two-level system and one photonic mode, known as the *quantum Rabi model* in the literature, whose Hamiltonian *Ĥ*_QRabi_ is given in eq 8. The other one is the slightly more general *Dicke model*, whose Hamiltonian *Ĥ*_Dicke_ is given by eq 23. The model consists of *N* two-level systems and a single QHO, and will be studied in selected parts of the article. These models have also gained interest in the mathematics community due to their quite intricate spectral properties.^42−44^ Recently, a quite advanced DFT approximation for the Dicke model was suggested by Novokreschenov et al.^45^ Finally, by performing the rotating-wave approximation, the Dicke and quantum Rabi models can be reduced to the Tavis–Cummings and Jaynes–Cummings models, respectively.

### 1.4 Elements from Standard Density-Functional Theory

Let us briefly recapitulate the mathematical description of standard DFT, in order to set the stage for our analysis of QEDFT. Standard DFT uses the one-body particle density ρ as the primary quantity to describe a system of *N* electrons within a quantum-mechanical treatment. Here, the density is defined from the wave function as
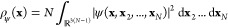
The set of fermionic *N*-particle wave functions considered are *L*^2^-normalized and have finite kinetic energy, which gives a set of *N*-representable densities (20) defined by the following properties,
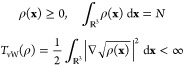
The set of *v*-representable densities is then the subset of densities ρ_ψ_ originating from ground states ψ of the Schrödinger equation with all possible external potentials *v*.^a^ The result that each *v*-representable density then corresponds to exactly one potential *v* (up to a constant) is the celebrated Hohenberg–Kohn theorem.^18^ Strictly speaking, further restrictions on the class of potentials considered are needed, before we can conclude that the potential is unique. We point the interested reader to Garrigue^46^ for further details. Perhaps even more important, the Hohenberg–Kohn theorem does not say anything about a possible surjectivity of the mapping from potentials to densities, i.e., it does not tell us which densities are *v*-representable in the first place.

Lieb’s^20^ explicit construction of a wave function from a determinant shows that for each ρ ∈  there is an antisymmetric wave function ψ that has ρ_ψ_ = ρ and finite kinetic energy. This is what the term *N*-representability for densities refers to. Then, Levy’s^47^ direct way of transitioning from a variation over wave functions to instead obtain the ground-state energy by means of a constrained search introduces the Levy–Lieb functional *F*_LL_,
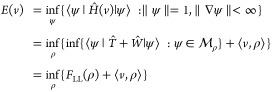
1Here *Ĥ*(*v*) = *T̂* + *Ŵ* + ∑_*i*=1_^*N*^*v*(**x**_*i*_) with *T̂* and *Ŵ* the standard kinetic energy and two-body operators, respectively, ⟨*v*, ρ⟩ =  and we introduced the constraint manifold
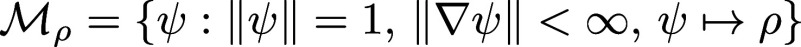
Equation 1 above defines the Levy–Lieb functional,
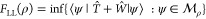
as a constrained minimization of the internal energy over . Its effective domain (the points where it is finite) is the set of *N*-representable densities . It is worth pointing out that even for *N*-representable densities, an optimizer ψ_0_ ∈  might not be associated with a Lagrange multiplier. In standard DFT, this Lagrange multiplier would be a scalar potential for which ψ_0_ is a ground state (or even an excited state). We bring this point up here for the benefit of the reader to contrast with the results available for the quantum Rabi and Dicke models of QEDFT presented later.

An alternative perspective on DFT is to view the ground-state energy and universal density functional as a conjugate (Legendre-)pair (*F,E*). In this spirit, Lieb introduced the *convex* functional
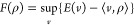
where *E*(*v*) is the ground-state energy introduced in eqs 1. It holds that *F* is the convex envelope of *F*_LL_ and that it is equal to the constrained-search functional over mixed (instead of pure) states.^20^ We can further replace *F*_LL_ by *F* in the expression for the ground-state energy, eq 1. Employing an unusual sign convention,^b^ (*F*,*E*) forms a conjugate pair, where a ground-state density ρ together with its potential *v* saturates the Fenchel–Young inequality *E*(*v*) – *F*(ρ) ≤ ⟨*v*, ρ⟩, i.e., *E*(*v*) – *F*(ρ) = ⟨*v*, ρ⟩. This *Lieb functional F* is defined on *X* = *L*^1^ ∩ *L*^3^, which properly contains , where *F*(ρ) = ∞ for all ρ that are not *N*-representable. In this mathematical setting, the natural space of potentials becomes X* = *L*^3/2^ + *L*^∞^, which includes molecular Coulomb-type potentials *v*(**x**) = ∑_*a*_*Z*_*a*_|**R**_*a*_ – **x**|^–1^. The potential space X* is the dual space of *X* and ensures finite interaction between the density ρ and the potential *v* by Hölder’s inequality,
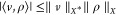


Placing our attention again on the variational principle for the ground-state energy, eq 1, we note that since the variation of ρ at any stationary point must be zero, we can equally formulate this with a differential. Then a potential *v* determined like this actually yields ρ in the ground state if we happen to be in a global minimum of ρ 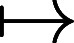
*F*_LL_(ρ) + ⟨*v*, ρ⟩, else it can still be the density of an excited state. But remember that we have *F*(ρ) convex, so using this functional instead, we can be sure that we obtain a potential that actually yields the correct density in the ground state. We thus write
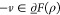
2where we used the generalized concept of a *subdifferential*, because *F*(ρ) is not differentiable in the usual sense.^48^ The subdifferential is defined as the set of all bounded tangent functionals below *F* at ρ,
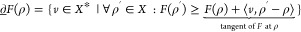
That is, if the subdifferential is nonempty then the potential is an element, else the density must be marked as non-*v*-representable. In standard DFT the potential is always unique by the Hohenberg–Kohn theorem (*if* it exists), so we have at most one element in the subdifferential ∂̲*F*(ρ) (up to adding a constant). This, however, is no longer true in other variants of DFT. Different potentials can lead to the same density variables and thus provide counterexamples to the Hohenberg–Kohn theorem on a finite lattice^49^ or in paramagnetic current-DFT,^50−52^ while the situation remains undecided in total-current DFT.^53^

Finally, we address the important exchange-correlation contribution *E*_xc_(ρ) to *F*(ρ). This can be given by the *density-fixed adiabatic connection*(54,55) that introduces a coupling constant λ in front of *Ŵ*. It is then possible to connect the simpler noninteracting case λ = 0 to the physical system of full interactions at λ = 1 (as dictated by *Ŵ*), or λ > 1 (or even taking λ → ∞, referred to as the strong-interaction limit.^56^) For a fixed ρ, this defines a λ-dependent Lieb functional *F*^λ^(ρ). We can then connect this functional at any λ > 0 to the noninteracting (kinetic energy only) *T*(ρ) = *F*^0^(ρ) by means of an integral representation with the Newton–Leibniz trick^57^
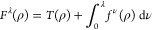
3Here we have used that λ 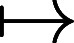
*F*^λ^(ρ) is a concave function and where *f*^ ν^(ρ) is any element of the *superdifferential* of λ 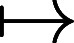
*F*^λ^(ρ). The superdifferential is the collection of all tangents that lie above the function, and is the concave equivalent of the subdifferential that we defined above. More precisely, in the current context the superdifferential is the set-valued mapping given by
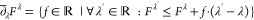
4We note in passing that the superdifferential is given by the interval [*a*,*b*] with *a* being the right and *b* the left derivative of *F*^λ^ at λ (and that those always exist and are equal except at countably many points). Assuming that the density matrix Γ^λ^ minimizes the internal energy from *T̂* + λ*Ŵ* under the given density constraint, it can be pointed out that^57^
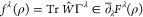
5Furthermore, we can subtract the Hartree energy, *E*_H_(ρ) = ∬ ρ(**r**)ρ(**r**′)/|**r** – **r**′| d^3^**r** d^3^**r**′, such that
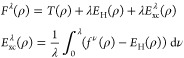
6If we set *E*_xc_(ρ) = *E*_xc_^λ=1^(ρ), we see that the adiabatic connection allows us to write (recall that Γ^λ^ is the minimizer corresponding to *F*^λ^(ρ))
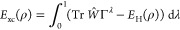
which is an important result in exact DFT for the unknown exchange-correlation contribution to *F*(ρ).

Furthermore, the term *E*_xc_ can itself be partitioned. The Levy–Perdew definition^58^ of the exchange energy as the high-density limit *E*_x_(ρ) = lim_γ→∞_ γ^–1^*E*_xc_(ρ_γ_) (where ρ_γ_(**r**) = γ^3^ρ(γ**r**)), then implies^57^ (from the fact that *F*^γλ^(ρ_γ_) = γ^2^*F*^λ^(ρ))
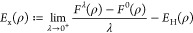
7In other words, the exchange energy is (after subtracting *E*_H_) the right derivative of the mapping λ 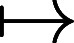
*F*^λ^(ρ) at λ = 0. The correlation term *E*_c_ is then simply the difference *E*_xc_ – *E*_x_.

Even from this short summary it becomes obvious that DFT has a very rich mathematical structure, but it is also ripe with difficulties such as non-*v*-representability and nondifferentiability.^48,59^ That is why it is beneficial, especially when including new effects, to study the extended theory thoroughly on the basis of simple model systems. It is thus the objective of this work to detail the above mathematical formulation of standard DFT in the context of model systems for QEDFT. Let us first turn to the QED-model Hamiltonians at hand, i.e., the quantum Rabi and Dicke models.

## 2 The Quantum Rabi Model

### 2.1 Model Definition

In atomic units,^c^ the Hamiltonian of the quantum Rabi model, *Ĥ*_QRabi_, from now on denoted *Ĥ*_0_, can be written as
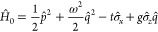
8It describes a single two-level system, like two levels in an atom or molecule, a simple dipole, or the spin states of an electron. This “matter” system gets coupled to a single photonic mode modeled as a quantum harmonic oscillator (QHO). Here ω is the frequency of the photonic mode, *t* > 0 the two-level kinetic hopping parameter, and *g* ∈  the coupling parameter between the photonic system and the two-level system. The operators
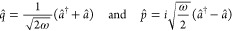
are the QHO position and momentum operators, respectively, and *â*^†^ (*â*) the raising (lowering) QHO ladder operators. Moreover,
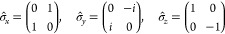
are the usual Pauli matrices.

**Figure 2:**
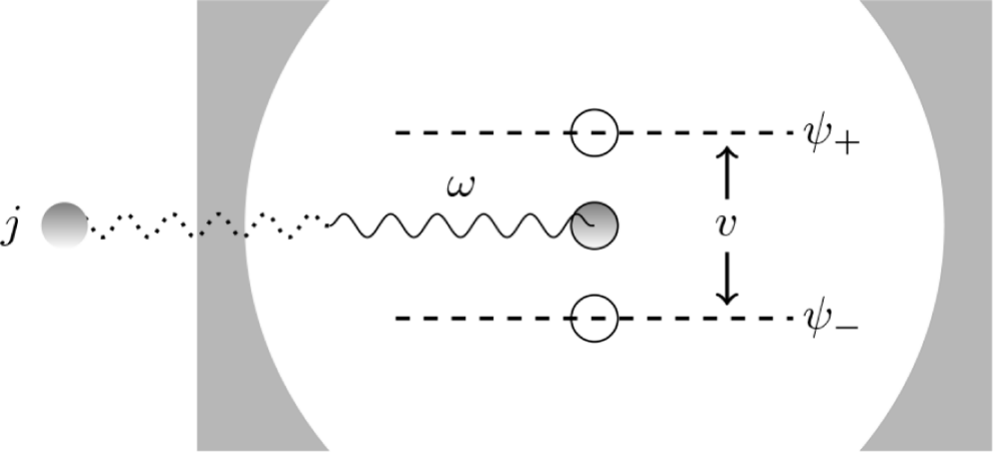
A schematic illustration of the full quantum Rabi model, eq 9, where ω is the photon frequency, the potential *v* tunes the level splitting, and *j* couples to the photonic degree-of-freedom. Here, ψ_±_ are the components of the wave function in the eigenbasis of σ̂_z_.

In order to be able to do DFT, we will consider an extension of the quantum Rabi model which couples the photonic mode and the two-level system to external quantities *j* ∈  and *v* ∈  respectively. We will refer to *Ĥ*_0_ as the *internal* Hamiltonian, whereas the *full* Hamiltonian is given by
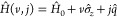
9The model setup of the full Hamiltonian is schematically illustrated in Figure 2. In this setting, applying a potential *v* corresponds to tuning the level splitting, whereas applying a current *j* displaces the QHO potential and induces an energy shift.^10^

### 2.2 Spaces and Domains

The state space of the system is the Hilbert space  =  ⊗  ≃ , allowing us to represent a general state  as a two-component function with respect to the eigenbasis of the σ̂_z_ Pauli operator,
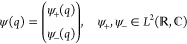
10We will work exclusively in this representation. The norm of ψ ∈  will be denoted by , and can be expressed in terms of the standard *L*^2^-norms of ψ_±_ as   =  + . The subscripts of the norms will be left implicit in the following. Likewise, the inner product between  can be expressed by the inner product in *L*^2^ between the components, ⟨ψ|φ⟩ = ⟨ψ_+_,φ_+_⟩ + ⟨ψ_–_,φ_–_⟩. We will thus reserve the notation ⟨·|·⟩ for inner products between states in the full Hilbert space of the system, and use ⟨·,·⟩ for inner products between the component wave functions in *L*^2^. The expectation value of an observable *Â* in the state ψ will be denoted ⟨ψ|*Â*|ψ⟩. We will usually leave the *q*-dependence implicit in integrals, and unless stated otherwise, all integrals are definite integrals over .

All physical eigenfunctions are subject to the usual constraint of finite energy. For just the QHO, this means
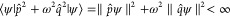
which is equivalent to ,  < ∞ holding simultaneously. These conditions give us a space for the *admissible* states, which is also the form domain^60^ of the operator *p̂*^2^ + ω^2^*q̂*^2^,
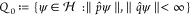
The Hamiltonians *Ĥ*_0_ and *Ĥ*(*v*,*j*) have a finite expectation value with respect to any ψ ∈ *Q*_0_.

We will now define two variables, σ_ψ_ and ξ_ψ_, that will later become important as descriptors of the system in the context of DFT. In particular, let us define the polarization
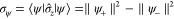
From the normalization condition ∥ψ∥ = 1, it follows that |σ_ψ_| ≤ 1 and that
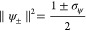
11Similarly, we define the displacement of the photon field as
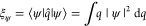
using the shorthand |ψ|^2^ = |ψ_+_|^2^ + |ψ_–_|^2^. We note that these variables satisfy (σ_ψ_,ξ_ψ_) ∈ [−1,1] ×  and we sometimes call them, in analogy to standard DFT, a *density pair*. They will be connected to the DFT treatment in Section 3.1, but for now they serve only as notational shorthands.

### 2.3 Properties of the Ground State

The point of departure for our investigation is the usual variational formulation for the ground-state energy corresponding to an external pair (*v*,*j*) ∈ ,
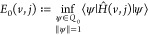
12Note that with the above definition alone, it is not guaranteed that there exists for all pairs (*v*,*j*) ∈  a normalized state in *Q*_0_ which realizes the ground-state energy *E*_0_(*v*,*j*), i.e., that the infimum is in fact a minimum. Nevertheless, in this section we will first obtain a lower bound on the energy and then establish the existence of a ground state that is analytic, real, strictly positive and unique.

We begin by noting that for any ψ ∈ *Q*_0_ the expectation value of the Hamiltonian is
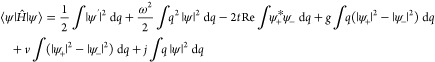
By completing the squares, we have the simpler form
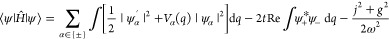
13where the harmonic-oscillator potentials are
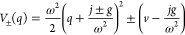
14and where the kinetic term
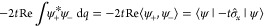
did not change. This shows that the quadratic form of *Ĥ*(*v*,*j*) can be expressed in terms of two coupled harmonic oscillators with potentials parametrically dependent on *v* and *j*. For the sake of brevity, let *C* = (*j*^2^ +*g*^2^)/2ω^2^. Using the Cauchy–Schwarz inequality, we obtain from eq 11 that the kinetic term fulfills the estimate
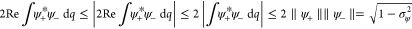
The Hamiltonian in eq 9 thus has a well-defined ground-state energy, *E*_0_(*v*, *j*), for all external pairs (*v*, *j*) ∈ , as the quadratic form of *Ĥ* is bounded from below,
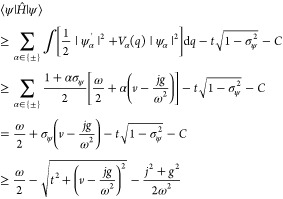
15Here, we have used eq 11 to attain the relative contributions of each shifted QHO to the energy, and the fact that the shifted QHO with potentials given by eq 14 independently have the energy solutions
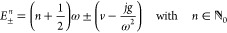
The final inequality can be shown by differentiating the preceding expression with respect to σ_ψ_, and setting the result to zero. The extremum is attained at
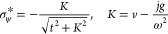
Note that |σ_ψ_^*^| < 1. Since the second derivative is positive for all σ_ψ_ ∈ (−1, 1) this extremum corresponds to a minimum, and it can easily be seen that this value is smaller than the value at the end points σ_ψ_ = ±1.

Together with the boundedness below, we can now use the knowledge that for potentials *V*_±_(*q*) → ∞ as |*q*| → ∞, as it is the case for the harmonic oscillator, the spectrum of the Hamiltonian is always discrete^61^ (this can also directly be argued from the compactness of the resolvent). This means that we always have a ground-state solution for any external pair (*v*, *j*) ∈ .

Next, we show that all eigenstates of the time-independent Schrödinger equation with Hamiltonian from eq 9 are analytic functions. Any eigenstate ψ with eigenvalue *E* is the solution to a system of second order ODEs:
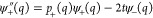
16a
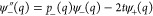
16b

The coefficients *p*_±_(*q*) are the polynomials in *q* given by
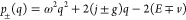
Setting ϕ_1_ = *ψ*_+_, ϕ_2_ = ψ_–_, ϕ_3_ = ψ_+_^′^, and ϕ_4_ = ψ_–_^′^ lets us express these equations as a system of first order ODEs
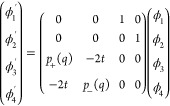
This setting shows that every solution to the time-independent Schrödinger equation is *analytic*, e.g., by Coddington and Carlson,^62^ Theorem 5.9 and Corollary 5.10.

For the real-valuedness of the components it is enough to see that the real and imaginary parts of ψ_±_(*q*) decouple in the expression of the quadratic form ⟨ψ|*Ĥ*|ψ⟩, and that the minimization can be carried out for the real and imaginary parts separately. This decoupling is readily apparent from eq 13 for all terms except −2*t*Re⟨ψ_+_,ψ_–_⟩. But for this term we have
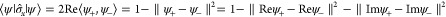
by the polarization identity, from which it is clear that the real and imaginary parts decouple for this term as well, meaning that ψ can be chosen to be real-valued.

Next, we demonstrate non-negativity. Let ψ be any admissible state and define the level sets *P*_±_ = {*q* ∈  : ψ_±_(*q*) ≥ 0} and *M*_±_ = {*q* ∈  : ψ_±_(*q*) < 0}. We can then construct the non-negative state
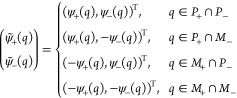
By direct calculation, we see that the constraints and all terms in ⟨ψ̃|*Ĥ*_0_|ψ̃⟩ are unchanged by the transformation ψ 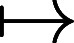 ψ̃, except the kinetic hopping term. However, by the polarization identity again
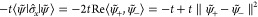
where the last term can be expanded as
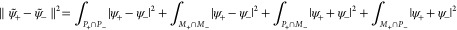
We then see that the two integrals on the first line remained unchanged by the transformation ψ 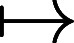 ψ̃, whereas the two integrals on the second line did not increase as their integrands contain a sum of a positive and a negative part. It then follows that the transformation ψ 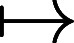 ψ̃ does *not* increase the energy and that ψ̃ must remain a ground state if ψ is one. We may thus conclude that the components of the ground state can always be chosen to be non-negative.

Now, take such an analytic and non-negative ground state and assume it has ψ_+_(*q*) = 0 at some *q* ∈ . Since it is non-negative it must hold ψ_+_^′^ = 0 and ψ_+_^″^ ≥ 0. Then by eq 16a, it follows from ψ_–_(*q*) ≥ 0 that ψ_+_^″^ ≤ 0, so actually ψ_+_^″^ = 0. But by the same equation this means also ψ_–_(*q*) = 0 and thus from non-negativity that ψ′_–_(*q*) = 0 and from eq 16b that ψ_–_^″^ = 0. But this just means that we have a zero solution since the initial values at *q* are zero, so we arrived at a contradiction. The argument for ψ_–_(*q*) = 0 is equivalent, so we can conclude that ψ(*q*) > 0 for all *q* ∈ . Analyticity also implies that the expectation values *σ*_ψ_ = ±1 that would rely on ψ_∓_ = 0 cannot be achieved by a ground state, nor by any other eigenstate, since eqs 16a and 16b show that this would imply a zero wave function.

Note that we carefully state that the components of the ground state “can be chosen” real and strictly positive, since we could not yet rule out the existence of other ground states that do not have this feature. However, the ground state of the quantum Rabi model must *always* be strictly positive by an argument that uses that the imaginary-time evolution is positivity improving.^63^ Now, since a second ground state, ψ_2_, must be orthogonal to the first one, ψ_1_, but is positive as well, so we inevitably get ⟨ψ_1_|ψ_2_⟩ > 0 which is a contradiction. We thus conclude that the ground state is also unique.

### 2.4 Results from the Hypervirial Theorem

With the system being fully defined by its Hamiltonian, eq 9, and having established some important properties of its ground state, we now move to deriving some useful relations between expectation values of operators with respect to any eigenstate (in particular the ground state). The hypervirial theorem offers a convenient tool for that task. The hypervirial theorem^64^ is the simple result that for any time-independent operator *Â* and an eigenstate ψ of *Ĥ* it holds by the Ehrenfest theorem (with τ the time variable)
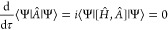
This is particularly useful if *Â* is chosen such that [*Ĥ*,*Â*] yields an operator of interest. This method was already applied to the Pauli–Fierz Hamiltonian to get field-mode virial theorems and expressions for the coupling energy.^65^ In the following, we will use the Heisenberg picture to give the time-derivative (first and sometimes second order) for a variety of operators that then give useful relations in the stationary setting. The calculation just requires the canonical commutator relations between *q̂* and *p̂* and the commutator relations between the Pauli matrices. For example, choosing *Â* = *q̂* gives
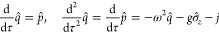
17For the expectation value, always with respect to an eigenstate ψ of *Ĥ*, we use the shorthand notation ⟨*Ô*⟩ = ⟨ψ|*Ô*|ψ⟩ in this section. Then the expectation values of the expressions above are
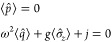
18Here, the last equation is the analogue of Maxwell’s equation in this reduced setting and connects ⟨*q̂*⟩ and ⟨*σ̂*_*z*_⟩ with the external *j*. This important relation will be used later multiple times. If we add the second order for *p̂*,
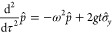
then since we already know ⟨*p̂*⟩ = 0, we get
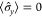
19This relation gets obvious if one writes it out in wave function components and takes into account that the ground-state wave function is always real (Section 2.3), yet we now additionally established that it holds for any eigenstate,
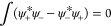


Another interesting choice is *Â* = *q̂p̂*,
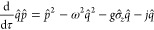
which leads to the usual virial theorem for the photon mode that relates to the coupling energy and the energy from current *j*,
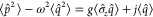
20Next, the Pauli operator σ̂_*z*_ yields the polarization as an expectation value,
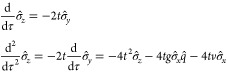
The expectation value of the first equation is just eq 19 again. However, the second equation allows us to determine the external potential *v* from expectation values via
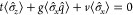
21and will thus be important for later applications. This expression is analogous to the force-balance equation that can also be employed in standard DFT and QEDFT to derive an exchange-correlation potential.^34,66^ This procedure will be showcased in Section 6.1 for the quantum Rabi model. Moreover, we give one last result from the hypervirial theorem of particular interest since it includes the coupling term,
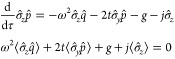
22Of course, many more such exact relations can be derived, but the above are the most significant for this study of the quantum Rabi model.

Before moving on to the analysis of the quantum Rabi model from a mathematical DFT perspective, let us briefly venture into its immediate extension. The reader should be forewarned that this section and Section 3.3 are somewhat peripheral to the main objectives of this article. However, it does provide some useful insights to notions not present in the quantum Rabi model, including a characterization of the set of densities where the Hohenberg–Kohn theorems hold.

### 2.5 The Dicke Model

Recall from our discussion of the model hierarchy in Section 1.3, see in particular Figure 1, that the immediate extensions of the quantum Rabi model are the Dicke model and its extension, the multimode Dicke model. For the sake of simplicity, let us only look at the Dicke model. However, the inclusion of multiple photonic modes does not significantly complicate the problem, and the interested reader is referred to Bakkestuen et al.^22^

The Dicke model describes a system of *N* two-level systems coupled to a single quantum harmonic oscillator. By extension of the state space for the quantum Rabi model, the state space of the Dicke model is the Hilbert space
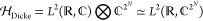
Thus, a state ψ ∈  has 4 components in the position basis if *N* = 2 and 8 components if *N* = 3. In analogy to eq 10 for *N* = 2 we write
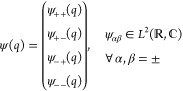
The appropriate inner product on , denoted in the same manner as for the quantum Rabi model, is then
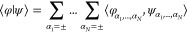
The displacement of a state is then
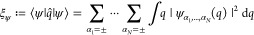


In order to define the polarization variable, we need to extend the definition of the Pauli operators to act on the Hilbert space of the Dicke model. For any 1 ≤ *j* ≤ *N*, the *lifted Pauli operators* are defined as
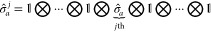
where *a* = *x*, *y*, *z*. By employing the usual matrix forms, the lifted Pauli-*z* matrices for *N* = 2 are
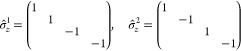
All the lifted Pauli matrices can then be collected into the vector **σ̂**_*a*_ = (σ̂_*a*_^1^, ..., σ̂_*a*_^*N*^)^T^ ∈ . Consequently, the polarization vector is given by
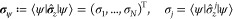
For *N* = 2, along with the normalization constraint, this yields
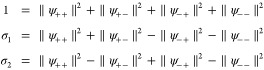
or alternatively
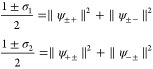
Equipped with the vector of the lifted Pauli operators, the internal *Dicke-model Hamiltonian* is
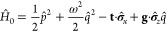
23where  is the kinetic hopping parameters and  the coupling parameters. For simplicity, the form domain of the Dicke Hamiltonian will also be denoted *Q*_0_. Coupled to the external quantities  and , the full Hamiltonian then is
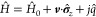
Despite having introduced the setting of the Dicke model, the following results, apart from Section 3.3 on the Hohenberg–Kohn theorem, will be mainly for the quantum Rabi model only.

## 3 Hohenberg–Kohn Theorems

### 3.1 Internal Variables for the Quantum Rabi Model

A common starting point for developing a DFT is to establish the connection between what are known as the internal and external variables of the system. As briefly mentioned in Section 1.4, the famous Hohenberg–Kohn theorem establishes that the internal variable—the electronic density—determines the external variable—the external potential—uniquely up to an additive constant. This mapping from the internal to the external variables firmly establishes the internal variable as a descriptor of the system (for a fixed number of electrons) in the case of Coulombic interactions.

Our point of departure to develop a QEDFT using the quantum Rabi model, and similarly for the Dicke model, will be to establish a Hohenberg–Kohn theorem. Recall from Section 2.2 that the full Hamiltonian is described by the pair of external quantities (*v*, *j*). Furthermore, we introduced two expectation values of particular interest,
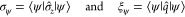
the polarization and the displacement of the photonic field, respectively. Moreover, in treating σ and ξ as free variables, not necessarily with a reference to a state, the previously given constraints demand σ ∈ [−1, 1] and ξ ∈ . The goal of this section is then to derive a Hohenberg–Kohn-type result, mapping a pair (σ,ξ) ∈ [−1, 1] ×  to an external pair (*v*, *j*) ∈  that yields exactly this density pair in the ground state. Deriving such a Hohenberg–Kohn-type result establishes the pair (σ, ξ) as the internal (density) variables of the system that fully determine the Hamiltonian at hand and thus also the ground state(s). This then sets the stage for developing a QEDFT using a Levy–Lieb functional in Section 4.

First, we show this for the quantum Rabi model. The proof strategy that we employ consists of separating the mapping from density pairs back to external pairs into two parts: First (HK1), the mapping from densities to ground states and second (HK2), the map from such states to external quantities. The reason is that while the first part is almost trivial and holds for all kinds of different DFTs, the second one is highly dependent on the system under consideration. This division of the Hohenberg–Kohn theorem is further detailed in Penz et al.^59^ Later, a Hohenberg–Kohn theorem for the Dicke model is also proven and we illustrate the notion of “regular” densities. Note also that the proof given for Theorem 3.6 is somewhat different from the proof given in Bakkestuen et al.,^22^ leading to an alternative characterization for regular densities.

### 3.2 Hohenberg–Kohn Theorem for the Quantum Rabi Model

The first part, also sometimes called the *weak Hohenberg–Kohn theorem*,^67^ shows that if two states have the same internal variables (σ, ξ) and they are ground states of Hamiltonians which differ only by the values of the external pair (*v,j*), then both states will also be ground states of the other Hamiltonian. We here use the notation ψ 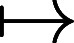 (σ,ξ) to state that a normalized ψ has σ_ψ_ = σ and ξ_ψ_ = ξ.

**Lemma 3.1**(Weak Hohenberg–Kohn, HK1). *Suppose* ψ_1_, ψ_2_ ∈ *Q*_0_*are ground states of Ĥ*(*v*_1_,*j*_1_) *and Ĥ*(*v*_2_,*j*_2_), *respectively. If both* ψ_1_,ψ_2_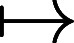 (σ, ξ), *then* ψ_2_*is a ground state of Ĥ*(*v*_1_,*j*_1_) *and* ψ_1_*is a ground state of Ĥ*(*v*_2_,*j*_2_).

In order to show this, first note that σ_ψ_1__ = σ_ψ_2__ = σ and ξ_ψ_1__ = ξ_ψ_2__ = ξ by assumption. Then it follows from eq 12 that any ground state ψ_*i*_ of *Ĥ*(*v*_*i*_, *j*_*i*_) is a minimizer of
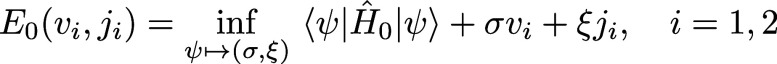
But *i* = 1 and *i* = 2 include exactly the same variational problem, such that any such minimizer ψ_*i*_ is a ground state for both *Ĥ*(*v*_1_, *j*_1_) and *Ĥ*(*v*_2_, *j*_2_).

Note that this type of argument works for any variant of DFT, as long as the external quantities couple only to the internal density variables.^59^ The second part of the Hohenberg–Kohn theorem, referred to as HK2, is then more geared to the special structure of the problem.

**Lemma 3.2**(HK2). *If two Hamiltonians Ĥ*(*v*_1_,*j*_1_) *andĤ* (*v*_2_,*j*_2_) *share any eigenstate then v*_1_ = *v*_2_*and j*_1_ = *j*_2_.

To prove this, let the two Hamiltonians *Ĥ*(*v*_1_, *j*_1_) and *Ĥ*(*v*_2_, *j*_2_) share the common eigenstate ψ ∈ *Q*_0_ and denote the respective eigenvalues by *E*_1_ and *E*_2_. Then, by the Schrödinger equation,
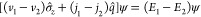
By assuming *j*_1_ ≠ *j*_2_, one obtains that
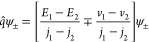
However, the operator *q̂* has no square-integrable eigenfunctions apart from the trivial solution ψ_±_ ≡ 0. Consequently, *j*_1_ = *j*_2_, since we of course cannot have ψ identically 0. This in turn implies that
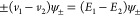
We can multiply these two equations by ψ_±_^*^ and integrate over *q* such that
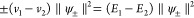
In this equation, we can also drop the squares on both sides and get
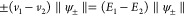
24(While this does not change anything here, we will need this form for the arguments of the next section.) Now, by what we know about eigenstates from Section 2.3, both components have ∥ψ_±_∥ ≠ 0 and the equations above give *v*_1_ – *v*_2_ = *E*_1_ – *E*_2_ = *v*_2_ – *v*_1_. So we must have *v*_1_ = *v*_2_, in which case also *E*_1_ = *E*_2_ and the Hamiltonians are identical.

A consequence of Lemma 3.2 is that the mapping from (*v*,*j*) to any eigenstate ψ of *Ĥ*(*v*,*j*) is injective. Since we already know that an eigenstate has always |σ_ψ_| < 1, we will remove the σ = ±1 from the following statement for the complete mapping (*v*,*j*) 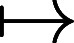 (σ,ξ). We will, as defined in Section 3.3 also for the more general case of the Dicke model, call the σ = ±1 critical, while σ ∈(−1, 1) are regular. If we combine the two results from Lemma 3.1 and Lemma 3.2 we get a full Hohenberg–Kohn theorem for the quantum Rabi model.

**Theorem 3.3** (Hohenberg–Kohn for the Quantum Rabi Model). *Any* (σ,ξ) ∈ (−1,1) × *that is the density pair of a ground state uniquely determines an external pair* (*v*,*j*) ∈ . *That is, the mapping*
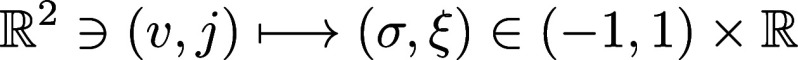
*is an injection.*

Note that unlike the original Hohenberg–Kohn theorem,^18^ Theorem 3.3 uniquely determines the external quantities, i.e., not only up to an additive constant. Then as noted in Section 3.1, Theorem 3.3 establishes the pair (σ, ξ) as the internal (density) variables of the system. The surjectivity of this mapping will be the result of the *v*-representability (actually (*v*, *j*)-representability, but we will stick to the usual DFT terminology here) of all regular density pairs (σ,ξ) ∈ (−1,1) ×  and will be demonstrated in Section 4.3.

### 3.3 Hohenberg–Kohn Theorem for the Dicke Model

In generalizing from the quantum Rabi model to the Dicke model, the Hohenberg–Kohn theorem is one of the complicating factors. To achieve it, as we will see in this section, we have to introduce the useful concept of a *regular* polarization vector **σ**. But first let us briefly state the analogous weak Hohenberg–Kohn result for the Dicke model.

**Lemma 3.4**. (Weak Hohenberg–Kohn, HK1). *Suppose that* ψ_1_, ψ_2_ ∈ *Q*_0_*are ground states of Ĥ*(***v***_1_,*j*_1_) *and Ĥ*(***v***_2_,*j*_2_) *respectively, where* (***v***_1_,*j*_1_) ∈  ×  and (***v***_2_,*j*_2_) ∈  × *are two external pairs. If both* ψ_1_,ψ_2_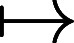 (**σ**,ξ), *then ψ*_2_*is a ground state of**and* ψ_1_*is a ground state ofĤ*(***v***_2_,*j*_2_).

The proof of this result is exactly the same as the proof of Lemma 3.1, and is thus a prime example of a case where the generalization from the quantum Rabi model to the Dicke model does not complicate matters. However, this is not entirely true for the generalization of Lemma 3.2, which requires the following definition of a regular polarization vector. Note that Bakkestuen et al.^22^ employ a different definition of regular **σ** that is adapted to the proof technique used there. However, these two definitions are equivalent.

**Definition 3.5** (Regular Polarization). A **σ** ∈ [−1,1]^*N*^ is called *regular* if for every χ ∈  that has ∥χ∥ = 1 and ⟨χ|**σ̂**_*z*_|χ⟩ = **σ** one has {χ, *σ̂*_*z*_^1^χ, ..., *σ̂*_*z*_^*N*^χ} as a set of linear independent vectors. The set of all regular **σ** is denoted . Any **σ** ∈ [−1,1]^*N*^ \  is not regular, and is called *critical*.

The relevance of this definition becomes immediately clear if we try to prove the second part of the Hohenberg–Kohn theorem for *N* ≥ 2 with the same method as in Lemma 3.2 before. We introduce χ ∈ , with components χ_α_1_,...,α_*n*__ = ∥ψ_α_1_,...,α_*n*__∥. Then ⟨χ|**σ̂**_*z*_|χ⟩ = ⟨ψ|***σ̂***_*z*_|ψ⟩ = **σ** and since the steps up to eq 24 are completely analogously we have
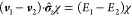
Here, each σ̂_*z*_^*j*^ in **σ̂**_*z*_ is purely diagonal and just applies the correct sign for every component of χ. Now assume that **σ** is a regular polarization vector, then all components χ, *σ̂*_*z*_^1^χ, ..., *σ̂*_*z*_^*N*^χ in the equation above are linearly independent and it directly follows ***v***_1_ – ***v***_2_ = 0 and *E*_1_ – *E*_2_ = 0 as required. This proves the full Hohenberg–Kohn theorem for all regular **σ** ∈ .

**Theorem 3.6** (Hohenberg–Kohn for the *N*-site Dicke Model). *Any* (**σ**,ξ) ∈  × *that is the density pair of a ground state uniquely determines an external pair* (***v***,*j*) ∈ . *That is, the mapping*
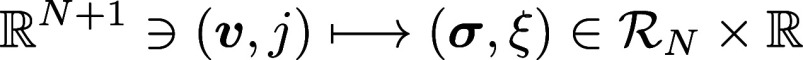
*is an injection.*

To exemplify the notion of regular polarizations, we will now show in three examples, for *N* = 1, 2, 3, how the set of critical polarization vectors looks like.

**Example 3.1** (*N* = 1). This is the case of the quantum Rabi model. Having **σ** ∈ [−1,1] regular demands that all χ ∈  with ∥χ_+_∥^2^ – ∥χ_–_∥^2^ = σ have {χ, σ̂_*z*_χ} linear independent. But (χ_+_, χ_–_) and (χ_+_, −χ_–_) are easily seen to be linear independent whenever both χ_±_ ≠ 0. This means the critical σ are those that come from χ_±_ = 0, exactly σ = ±1. The regular set is then  = (−1,1).

**Figure 3:**
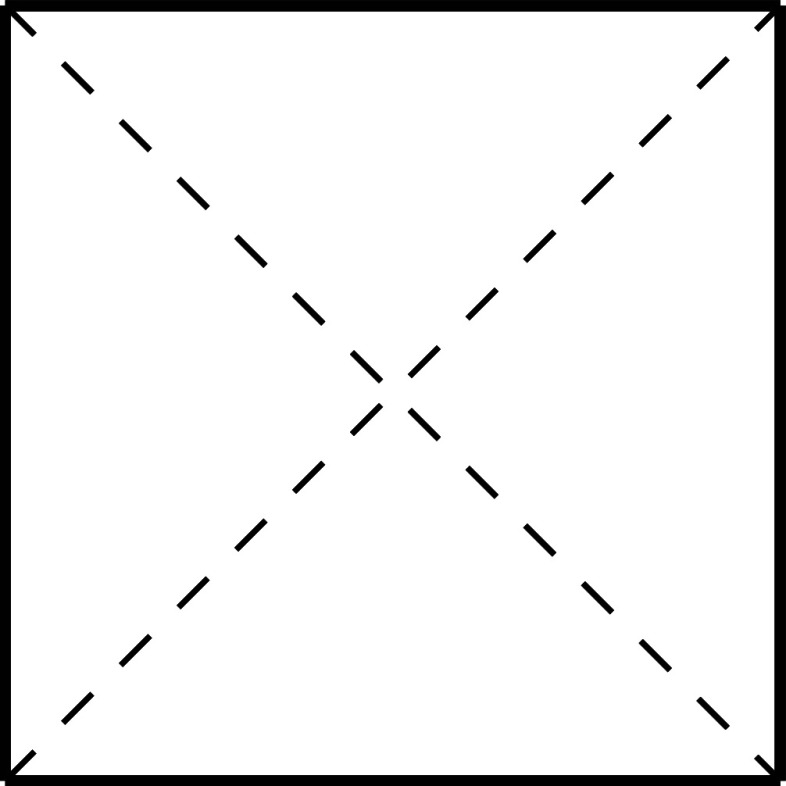
Set of regular densities for *N* = 2,  ⊂ (−1,1)^2^, is the union of four congruent open triangles.

**Example 3.2** (*N* = 2). Now χ ∈  and we need to have the three vectors {χ, σ̂_*z*_^1^χ, σ̂_*z*_^2^χ} linear independent. Instead of writing things out in components, we will argue combinatorially. Three out of four nonzero components are enough to have the three vectors linearly independent, consequently we get an critical **σ** from χ whenever two or three components vanish. Three vanishing components just leaves four possibilities, χ_1_ = (1, 0, 0, 0)^T^ and its permutations χ_2_, χ_3_, χ_4_, which map to **σ** = [±1,±1], precisely the four corners of the polarization square [−1,1]^2^. A linear combination of two χ_*i*_ still has two zero-components and maps to the straight lines that connect two corners. We thus have
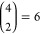
such lines that represent critical polarizations. The situation is depicted in Figure 3.

**Figure 4:**
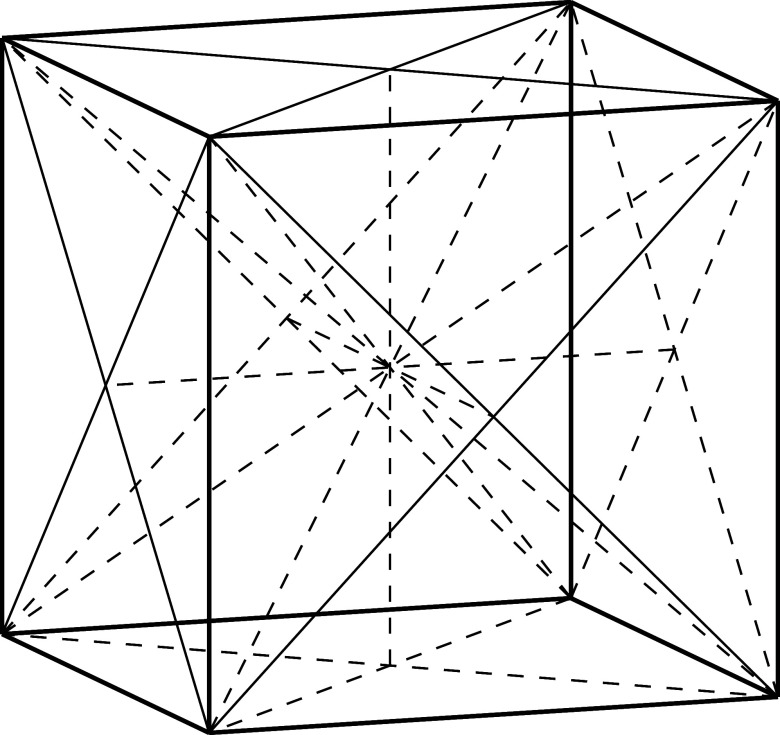
Set of regular polarizations for *N* = 3,  ⊂ (−1,1)^3^, is the union of open polyhedra created by 14 planes cutting through the open cube (−1,1)^3^. These are the 6 planes extending from the diagonals on the faces plus the 8 planes formed by taking three face-diagonals.

**Example 3.3** (*N* = 3). We have χ ∈  and argue as before. A critical **σ** comes from a χ that has between five and seven zero-components. On the other hand, four nonzero components are enough to have {χ, σ̂_*z*_^1^χ, σ̂_*z*_^2^χ, σ̂_*z*_^3^χ} linear independent. If χ has just one nonzero component, it maps to the 8 corners of the polarization cube [−1,1]^3^. The
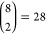
ways how to combine these corners then come from χ with two nonzero components. These are the 12 edges of the cube, plus 12 diagonals on the faces, plus 4 diagonals in the inside of the cube. Finally, we always take three of the corners of the cube and combine them into
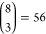
planes that belong to χ with three nonzero components. Here one sees that many of these combinations actually yield the same plane, so that only 20 remain: 6 faces, 6 from the diagonals on the faces connecting to the opposite face, and 8 that are formed by three face-diagonals. The situation is depicted in Figure 4.

Already from the above examples up to *N* = 3 one realizes that for general *N*, the regular set  is the union of disjoint open convex polytopes.^22^ In *N* = 2 the polytopes are simply triangles, while in *N* = 3 they are polyhedra.

In summary, given a ground-state density pair (**σ**, ξ), the uniqueness for external pairs can only be guaranteed for regular **σ**. Nevertheless, we never encountered a nonunique external pair in case of the Dicke model, in contrast to lattice models, where explicit counterexamples to a full Hohenberg–Kohn theorem are known.^49^ Note especially that such counterexamples always rely on ground-state degeneracy,^68^ but such a degeneracy was also never observed in our numerical investigations of the Dicke model up to now.

## 4 The Levy–Lieb Functional

### 4.1 Definition

From the investigation of the Hohenberg–Kohn theorems in the preceding section, the internal variables σ ∈ [−1,1] and ξ ∈  arise as descriptors of the system. This justifies, in analogy to standard DFT, referring to them collectively as a density pair. Consider now the minimization of the internal energy ψ 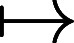 ⟨ψ|*Ĥ*_0_|ψ⟩ under the constraint of normalizable and admissible ψ mapping to the correct density pair, i.e., σ_ψ_ = σ and *ξ*_ψ_ = *ξ*. This problem naturally gives rise to what is commonly called the *pure-state constrained-search functional*, also known as the *Levy–Lieb functional*, introduced in Section 1.4 for standard DFT. However, before defining this functional for the quantum Rabi model, let us turn to the question of whether a given density pair (σ,ξ) ∈ [−1,1] ×  can even be represented by a state ψ.

**Theorem 4.1**. (*N*-representability). *For every density pair* (σ,ξ) ∈ [−1,1] × *there exists* ψ ∈ *Q*_0_*such that* ∥ψ∥ = 1, ⟨ψ|*σ̂*_*z*_|ψ⟩ = σ, and ⟨ψ|*q̂*|ψ⟩ = ξ.

Note here, that “*N*-representability” is a technical term from the standard DFT terminology and just means that the given constraints can be fulfilled by an *N*-particle state. In the quantum Rabi model, naturally, this *N* has no further meaning. However, Theorem 4.1 also holds for the Dicke model, in which case the *N* is not to be confused with the number of two-level systems. We give a simple and constructive proof.

*Proof of Theorem 4.1*. Let us fix a density pair (σ,ξ) ∈ [−1,1] ×  and suppose the Gaussian trial state
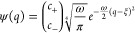
25Choosing *c*_±_ =  then satisfies all the constraints.

The state of eq 25 is not only the prototype for a state with (σ_ψ_, ξ_ψ_) = (*σ*, *ξ*) but will also reappear as the optimizer for critical σ and at zero coupling.

Motivated by Theorem 4.1, let us introduce the *constraint manifold*   collecting all normalized and admissible states that map to a given density pair (σ,ξ) ∈ [−1,1] × ,
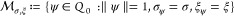
Then using Theorem 4.1 and eq 9 we may for any (*v*,*j*) ∈  perform the following partitioning,
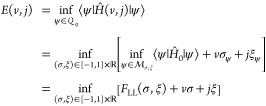
26This expression defines the *Levy–Lieb functionalF*_LL_: [−1,1] ×  →  by
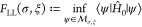
27It then immediately follows that |*F*_LL_(σ, ξ)| < ∞. The boundedness below was shown in eq 15, while the boundedness above is clear from the existence of a trial state. Moreover, for the quantum Rabi model we can prove a wide range of additional properties of *F*_LL_ and its optimizers.

### 4.2 Properties of the Levy–Lieb Functional

We summarize our results in the following theorem.

**Theorem 4.2**. *For every density pair* (σ,ξ) ∈ [−1,1] × *and any optimizer ψ* of *F*_LL_(σ, ξ), *the following properties are satisfied.*

1 *F*_LL_(σ, ξ) = *F*_LL_(−σ, −ξ).
2 *For any* ζ ∈ , *the displacement rule*
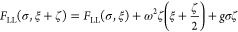
*holds, with the special case ζ* = 0,
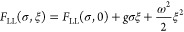
3 *An optimizer can always be chosen real and non-negative in both components* (ψ_±_).
4 *Any optimizer* ψ *of F*_LL_(*σ*, 0) *satisfies the virial relation*
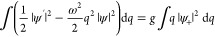
5 *For any optimizer*
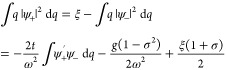
6 *Any optimizer satisfies the bound*
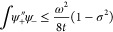

Let us now prove these properties and make some remarks on their relevance. For the sake of readability, the proof of Theorem 4.2 has been divided up into five smaller sections including the respective discussions.

#### 4.2.1 Proof of Theorem 4.2.1: Symmetry of the Levy–Lieb Functional

Take the joint transformation of parity and time conjugation^d^, i.e., ψ_±_(*q*) 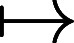 ψ̃_±_(*q*) = ψ_∓_(*q*). The  transformation is clearly a unitary transformation that yields
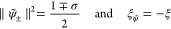
thus implying that 
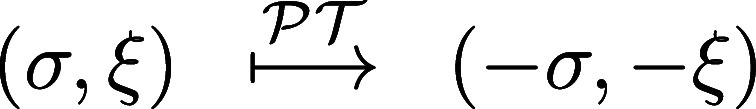
Furthermore, the transformation leaves the quadratic form of the internal Hamiltonian unchanged, i.e., ⟨ψ̃|*Ĥ*_0_|ψ̃⟩ = ⟨ψ|*Ĥ*_0_|ψ⟩. It then follows that the Levy–Lieb functional is *symmetric* in the density pair (σ, ξ) for all possible pairs, which concludes the proof.

This symmetry simplifies further studies of the functional, in particular since Theorem 4.2.2 explicitly determines all dependence in the displacement, ξ. The symmetry thus implies that it will always be sufficient to investigate the functional for σ ∈ [0, 1] at one fixed value of ξ, typically chosen to be zero. This is especially useful in the numerical investigations, as it effectively halves the search space in σ.

#### 4.2.2 Proof of Theorem 4.2.2: Displacement of the Levy–Lieb Functional

Consider the shift operator  which displaces the harmonic-oscillator coordinate by ζ ∈ . In particular,  maps ψ_±_(*q*) to ψ_±_(*q* – ζ), while leaving the two-level decomposition unchanged. It then follows that  = ∥ψ∥^2^,  = σ_ψ_, and  = ξ_ψ_ + ζ. From a direct computation we have
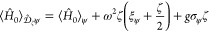
Then, from the definition of the Levy–Lieb functional we obtain
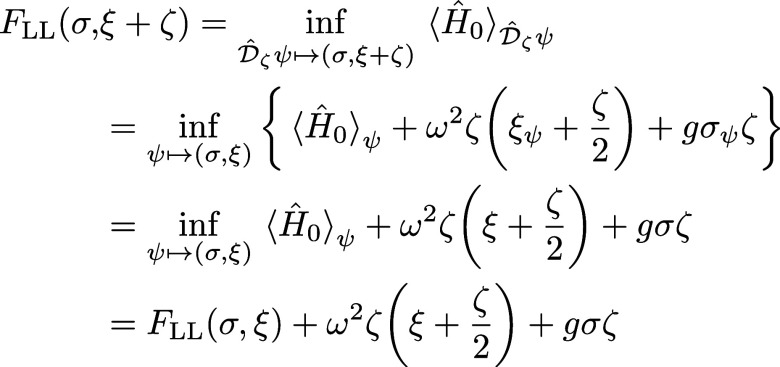
which is the general displacement relation. Note the second to last equality, in which we restrict the minimization to apply to the first term only. The search for minimizers is performed over the space of wave functions which map to a specific set of internal variables (σ, ξ), which means that all terms but the first are constant during the minimization process. Further note that the above also shows that if ψ is the optimizer of *F*_LL_(σ, ξ) then  is the optimizer of *F*_LL_(σ, ξ + ζ). Thus, if the optimizer is known at one ξ, all other optimizers in ξ can be obtained by displacement. Unfortunately, the dependence in the polarization is not as simple.

The result in Theorem 4.2.2, which also holds in the generalization to the Dicke model, is important as it always allows us to explicitly extract all dependency on the displacement ξ.

#### 4.2.3 Proof of Theorem 4.2.3: Real and Positive Optimizers

The statement follows from the same argument as the real-valuedness and non-negativity of the components of the ground state given in Section 2.3. In particular, the argument also holds for optimizers corresponding to the critical points σ = ±1.

While the real-valuedness also holds for the Dicke model,^22^ the non-negativity does not straightforwardly follow. This is important, since the result that optimizers of the Levy–Lieb functional are always ground states (Theorem 4.7) relies on this feature.

#### 4.2.4 Proof of Theorem 4.2.4: Virial Relation

Let us consider the usual an-isotropic coordinate scaling of Levy and Perdew,^58^ that is scaling the harmonic-oscillator coordinate only. For μ > 0 define the transformation ψ 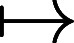 ψ^μ^ of an optimizer by ψ_±_^μ^(*q*) = ψ_±_(μ*q*). By direct calculation we find that
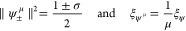
and also that
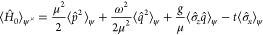
Let ψ be an optimizer of *F*_LL_(*σ*, 0), then σ = σ_ψ_ = σ_ψ^μ^_ and ξ = ξ_ψ_ = ξ_ψ^μ^_ = 0 are left unchanged by the scaling. The stationarity condition on the internal energy
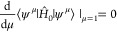
implies that
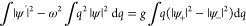
Then, if we recall that ξ_ψ_ = ∫*q*(|ψ_+_|^2^ + |ψ_–_|^2^)d*q* = 0, we obtain that
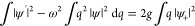
from which Theorem 4.2.4 follows. The same result was already achieved in eq 20 in the context of the hypervirial theorem. This virial relation will be of importance when analyzing the adiabatic connection for the Levy–Lieb functional in Section 5.

#### 4.2.5 Proofs of Theorem 4.2.(5–6): Relations for the Kinetic Hopping

Given an optimizer ψ, consider the transformation ψ 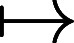 ψ^*s*^ for some *s* ∈  and fixed σ ∈ [−1,1] given by
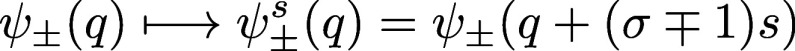
It then follows from direct calculation that the transformation leaves the constraints unchanged, in particular σ_ψ^s^_ = σ_ψ_ = σ and ξ_ψ^*s*^_ = ξ_ψ_ = ξ. Let us then consider ⟨*Ĥ*_0_⟩_ψ^*s*^_, the expectation value of the internal Hamiltonian with respect to the state ψ^*s*^, which contains the following terms
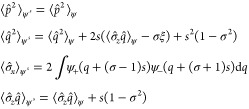
where we already used the result from Theorem 4.2.3 that the optimizer can be chosen real. The stationarity condition
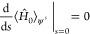
then implies that
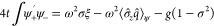
using integration by parts. Then, using again that ∫*q*(|ψ_+_|^2^ + |ψ_–_|^2^)d*q* = ξ, we have that ⟨σ̂_*z*_*q̂*⟩_ψ_ = 2∫*q*|ψ_+_|^2^ d*q* – ξ such that
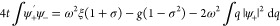
Theorem 4.2.5 then follows from a slight rearrangement. Note that the same result also follows from using eqs 22 and 18 together.

Finally, in order to show Theorem 4.2.6, we consider the second-order condition
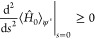
Then only two terms will contribute, and it follows from integration by parts that
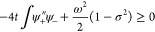


### 4.3 Optimizers are Ground States and *v*-Representability

In this section we discuss the relation between optimizers of the Levy–Lieb functional and eigenstates for the Schrödinger equation of the quantum Rabi model. Importantly, we will show that optimizers are even ground states and show full *v*-representability if . But first, the question arises whether the infimum can always be attained, i.e., if an optimizer even exists. Fortunately, this question can be answered positively.

**Theorem 4.3***For every density pair* (σ,ξ) ∈ [−1,1] × , *there exists an optimizer* ψ ∈ *such that*
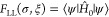
*i.e., the infimum in eq 27is a minimum.*

The proof, also for the more general case of the Dicke model, can be found in Bakkestuen et al.,^22^ Theorem 3.4. Note that within the standard formulation of DFT, the proof of the analogous statement, e.g., Lieb,^20^ Theorem 3.3, relies on the density constraint on the wave function to obtain the necessary convergence of the optimizing sequence.^e^ In the setting of the quantum Rabi model and the Dicke model, this feature can be obtained by the trapping nature of the harmonic-oscillator potential.

For regular values of σ, the following optimality result is obtained in the language of Lagrange multipliers by use of the first and second order criticality conditions on the quadratic form ⟨ψ|*Ĥ*_0_|ψ⟩. The use of Lagrange multipliers comes naturally, since we have three constraints to fulfill within the variational problem of eq 27: ∥ψ∥ = 1, σ_ψ_ = σ, and ξ_ψ_ = ξ. The corresponding Lagrange multipliers are then *E*, *v*, and *j*. For the full argument, see the proof of Theorem 3.7 in Bakkestuen et al.,^22^ where the result is shown to hold for all regular σ in the generalization to the Dicke model, or Theorem 3.18 in the same reference for a specialization to the quantum Rabi model.

**Theorem 4.4***Let* (σ,ξ) ∈ (−1,1) × *and suppose that* ψ ∈ *is an optimizer of F*_LL_(σ, ξ). *Then there exist unique Lagrange multipliersE*, *v*, *j* ∈ *such that* ψ *satisfies the Schrödinger equation (in the strong sense)*
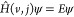
28*as well as the second-order condition*
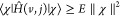
*for all χ in the tangent space of* at ψ, *characterized by* χ ∈ *Q*_0_, ⟨ψ|χ⟩ = 0 , ⟨ψ|σ̂_*z*_|χ⟩ = 0, and ⟨ψ|*q̂*_*z*_|χ⟩ = 0. *Furthermore,* ψ *has the internal energy*
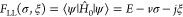


**Figure 5:**
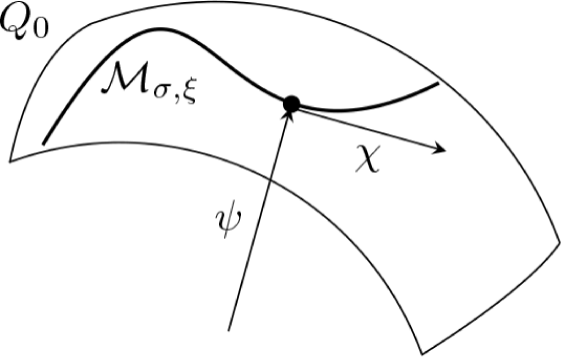
Constraint manifold  inside the set of admissible wave functions *Q*_0_ with an optimizer ψ and a vector χ from the tangent space.

The situation is sketched in Figure 5. Note that while a constraint manifold can be analogously formulated within the standard formulation of DFT, the full result from above cannot be obtained since the density constraint does not give rise to a well-defined tangent space due to problems with differentiability.^48^

Additionally, a similar result can also be formulated for the critical values of σ, which in the case of the quantum Rabi model are simply the end points σ = ±1.

**Theorem 4.5***Let* σ = ±1 and ξ ∈ . *Suppose that* ψ ∈ *is an optimizer of F*_LL_(σ, ξ). *Then* ψ_∓_ ≡ 0 *and there exists an n* ∈ *such that* ψ̃_±_(*q*) = ψ_±_(*q* + ξ) *satisfies the harmonic-oscillator equation*
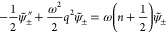
29*in the strong sense. The ψ has the internal energy*
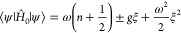
30

The proof of this results relies, similarly to the proof of Theorem 4.4, on the criticality condition of the quadratic form ⟨ψ|*Ĥ*_0_|ψ⟩. The full derivation can be found in Bakkestuen et al.,^22^ Theorem 3.18, but note here that this result does not immediately generalize to the Dicke model. In fact, the optimality conditions for the critical σ’s can also be written down for the general Dicke model, but they are significantly more complicated and do not take the simple form of eqs 29 and 30. Moreover, the following result follows immediately from Theorem 4.5, in particular from the harmonic-oscillator solution of eq 29 where, quite obviously, we can limit ourselves to *n* = 0 for the optimizer that is thus unique.

**Corollary 4.6***For any* ξ ∈ *and critical σ*, *the Gaussian trial state from**eq*25, *withc*_+_ = 1, *c*_–_ = 0 *if* σ = +1 *andc*_+_ = 0, *c*_–_ = 1 if σ = −1, *is the unique optimizer ofF*_LL_(±1,ξ). *The corresponding values of the Levy–Lieb functional are*
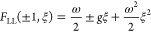


Can we say something similar for the regular values of σ? Indeed the optimizers of *F*_LL_(σ, ξ) are always unique and not only solve the Schrödinger equation but constitute its *ground-state solution*. This important result does unfortunately not generalize fully to the Dicke model. There, we may only conclude that the optimizer is a low-lying eigenstate, in particular at most the (*N* + 1)-th lowest eigenstate (see Bakkestuen et al.,^22^ Theorem 3.9).

**Theorem 4.7***For every density pair* (σ,ξ) ∈ (−1,1) × *there exists a unique real-valued and strictly positive optimizer* ψ ∈  of *F*_LL_(σ, ξ) *that is the (nondegenerate) ground-state solution of eq 28* .

*Proof.* First, we invoke Theorem 4.3 to show that an optimizer ψ exists and Theorem 4.2.3 to have it real and with non-negative components. Furthermore, the optimizer ψ satisfies the Schrödinger equation in the strong sense by Theorem 4.4. This means it has all the properties that we showed for ground states in Section 2.3, in particular that it is unique.

The preceding theorem has tremendous consequences. First, since every regular density pair is a ground-state solution for an Hamiltonian with some (*v*,*j*) ∈ , we achieved a full *v*-representability result. Second, since this ground state is always nondegenerate, there is no need to ever rely on mixed states. This means that the Lieb functional *F*_L_,^20^ that is equal to the constrained search over ensembles, will give the same result. In other words, the Levy–Lieb functional and the Lieb functional are the same. Finally, since the Lieb functional is always convex, it implies that *F*_LL_ is also convex. Then since the external quantities (*v*, *j*) from the subdifferential −∂̲*F*(σ, ξ) are unique as Lagrange multipliers (Theorem 4.4), the functional is differentiable by a basic result from convex analysis (see, e.g., Theorem 25.1 in Rockafellar.^71^ We collect those results in the following corollary.

**Corollary 4.8***Consider a regular density pair* (σ, ξ) ∈  (−1,1) × . *Then the following holds:*

1 (*v*-*representability*) *The* (σ, ξ) *is uniquely pure-state v*-*representable*.
2 (*equivalence of functionals*) *F*_LL_(σ, ξ) = *F*_L_(σ, ξ).
3 (*differentiability*) *The F*_LL_*is differentiable at* (σ, ξ) *and* (*v*, *j*) = −∇*F*_LL_(σ, ξ) *is its representing external potential pair*.

Here, Item 1 shows that the mapping (−1,1) ×  ∋ (σ, ξ) 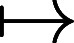 (*v*,*j*) ∈  is a bijection. This already includes the Hohenberg–Kohn result of injectivity from Theorem 3.3. Yet, if σ = ±1 then (σ, ξ) is not *v*-representable. This is also a significantly stronger result than for other settings where *v*-representability is not available. Item 2 also holds for the critical end points at σ = ±1, where the proof, alongside a more detailed proof also for the other points, can be found in Bakkestuen et al.^22^ For |σ| → 1 the derivative from Item 3 must go to infinity, |*v*| → ∞, since the derivative of a convex function must always be monotone.

### 4.4 The Levy–Lieb Functional at Zero Coupling

For the purposes of the later discussion on the adiabatic connection in Section 5, let us consider the special case of *g* = 0. In analogy to the usual quantum chemistry language this can also be referred to as the Kohn–Sham model of the system as it describes a noninteracting (or rather uncoupled) variant of the model at hand. In this case the internal Hamiltonian simplifies to
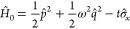
31which then naturally is diagonal in the eigenbasis of σ̂_*x*_.

Recall from Section 2.3 that we may approximate the expectation value of eq 31 from below by the ground state of the harmonic oscillator and estimate the kinetic coupling term by the Cauchy–Schwarz inequality, such that
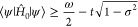
This is of course a lower bound on *F*_LL_^0^ as well, in particular on *F*_LL_^0^(σ,0). Note that we use a zero superscript to denote the Levy–Lieb functional in the special case of *g* = 0. Then by the displacement rule, Theorem 4.2.2,
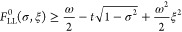
32

Suppose again the Gaussian trial state, eq 25, with the coefficients *c*_±_ =  which as previously shown satisfies the constraints. For this particular choice of trial state, ψ, we find that
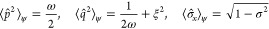
Combining this with the constraints, we find that the trial state has the internal energy
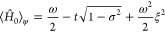
which would be an upper bound on the ground state energy if the trial state is not a ground-state. However, it is in fact exactly equal the lower bound eq 32, thus implying that the trial state, eq 25, is indeed the optimizer of the Levy–Lieb functional at zero coupling, and by Theorem 4.7 we can formulate the following result.

**Theorem 4.9** (Optimizer at Zero Coupling). *For g* = 0 and (σ,ξ) ∈ [−1,1] × , *the Gaussian trial state, eq*25, *with coefficientsc*_±_ = *is the optimizer of the Levy–Lieb functional. For all pairs* (σ,ξ) ∈ [−1,1] × *it holds*
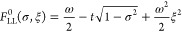


This result will be of importance when we turn to the discussion of the adiabatic connection of the Levy–Lieb functional in Section 5.

**Figure 6:**
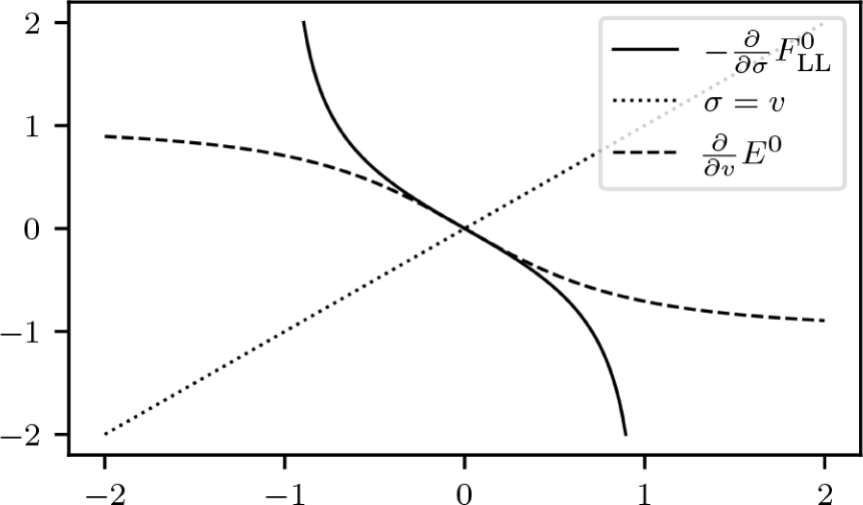
At λ = 0 we can explicitly illustrate how *F*_LL_^0^ and *E*^0^ encode the same information, in a plot inspired by the work of Helgaker and Teale.^37^ Reflection of the differential  along σ = *v* allows us to obtain .

Furthermore, since the value of the Levy–Lieb functional is explicitly known in the zero coupling regime, it allows us to calculate the ground-state energy directly using eq 26. By virtue of the unique *v*-representability, Corollary 4.8.1, the infimum in eq 26 is indeed a minimum for all regular densities such that the energy can be readily calculated as direct minimization over (σ,ξ) ∈ (−1,1) × . However, as noted in Corollary 4.8.3, the functional is differentiable for regular polarizations. Therefore, we may for each (σ,ξ) ∈ [−1,1] ×  calculate the corresponding external pair directly as the derivative of *F*_LL_^0^(σ,ξ). In particular,
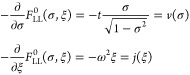
These expressions define the explicit one-to-one Hohenberg–Kohn mapping in this simplified setting. Inverting these expressions, the optimizers of eq 26 are
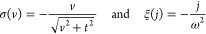
Here, the expression for the displacement is the virial relation eq 18 again, in the special case of *g* = 0. By insertion into eq 26 we have
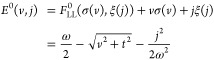
Note that for the zero-coupling conjugate pair (*F*_LL_^0^,*E*^0^), the Fenchel–Young inequality *E*^0^ – *F*_LL_^0^ ≤ *v*σ + *j*ξ is then fully saturated at the optimizers, as required. This is also directly seen to be true for the extended version of *F*_LL_^0^ defined on the whole  by setting the functional value equal to +∞ whenever |σ| > 1 (i.e., when a density pair is non *N*-representable) or using the Lieb recipe
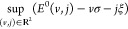
for every (σ,ξ) ∈ . We can make one more comment within the framework of convex analysis. For a conjugate pair like (*F*_LL_^0^,*E*^0^), we know that the optimality condition can be expressed as^f^
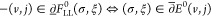
The same information is encoded in *F*_LL_^0^ and *E*^0^. In this simplified setting we can interpret the calculations above that took us from *F*_LL_^0^ to *E*^0^: by first computing the differentials −∂̲*F*_LL_^0^ we can invert these expressions, or said differently, reflect the expressions along σ = *v* and ξ = *j*. This mirroring operations give the elements of ∂̅*E*^0^ geometrically. Integrating these differentials takes us to the energy expression *E*^0^ (up to a constant). In the case of the extended universal functional, that assumes the value +∞, the vertical asymptotes of the differentials are reflected to horizontal ones. The process is illustrated in Figure 6. In summary, the zero coupling case, that will paradigmatically serve as a Kohn–Sham system, thus allows for an explicit form for the mapping to external pairs, the ground-state energy, and the universal functional.

## 5 The Adiabatic Connection

### 5.1 Integral Representation of the Universal Functional

For the study of the adiabatic connection within this DFT formulation of the quantum Rabi model, let us introduce λ ∈  as a scaling for the coupling constant in a similar fashion as in standard DFT, discussed in Section 1.4. In this section, let us indicate the dependence on the λ by a superscript, which for the internal Hamiltonian entails,
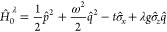
The Levy–Lieb functional, denoted *F*_LL_^λ^(σ,ξ), is then given by (for a given λ)
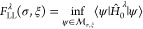
Thus, λ = 0 gives the uncoupled system while λ = 1 corresponds to the previously considered Hamiltonian with light-matter coupling. Moreover, to show that the function 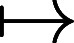
*F*_LL_^λ^(σ,ξ) is concave for every fixed pair (σ,ξ) ∈ [−1,1] × , choose some λ_1_,λ_2_ ∈  and *s* ∈ [0,1]. It then follows that
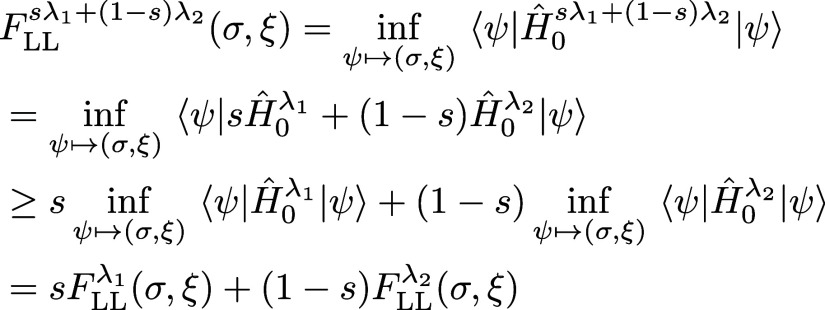


To obtain the adiabatic connection, we will first calculate the superdifferential of the function λ 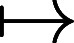
*F*_LL_^λ^(σ,ξ) that we defined in eq 4. Let ψ^λ^ ∈  be the optimizer of *F*_LL_^λ^(σ,ξ) and ψ^λ^′^^ ∈  be the optimizer of *F*_LL_^λ^′^^(σ,ξ). It then follows from the variational principle that
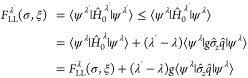
We thus have the following result that echoes the standard DFT result from eq 5.

**Lemma 5.1***For every fixed pair* (σ,ξ) ∈ [−1,1] × *and* λ ∈ 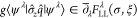
*where* ψ^λ^ ∈ *is the unique real-valued and strictly positive optimizer ofF*_LL_^λ^(σ,ξ).

We have been careful here not to assume that λ 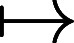
*F*_LL_^λ^(σ,ξ) is differentiable, but actually the nondegeneracy of ground states and the differentiability of *F*_LL_^λ^(σ,ξ) with respect to σ and ξ from Corollary 4.8.3 is a strong indication that the functional is also differentiable with respect to λ.

Before applying the Newton–Leibniz formula, eq 3, to obtain an integral representation of *F*_LL_^λ^, let us first employ the displacement rule, Theorem 4.2.2,
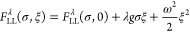
The Newton–Leibniz formula applied to *F*_LL_^λ^(σ,0) using Lemma 5.1 as the choice for the element of the superdifferential then yields
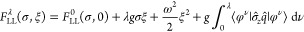
33where φ^*ν*^ ∈  denotes the optimizer of *F*_LL_^λ^(σ,0). Importantly, as the φ^*ν*^ do not depend on ξ, the above integrand is also independent of ξ. Moreover, recall from Theorem 4.2.5 that the integrand in eq 33 can be rewritten as
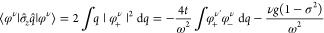
Using this form, along with the explicit expression *F*_LL_^λ^(σ,0) = ω/2 – from Theorem 4.9, we obtain the following result.

**Theorem 5.2***For every* λ ∈ *the Levy–Lieb functional along the adiabatic connection, F*_LL_^λ^: [−1,1] ×  → , *is given by*
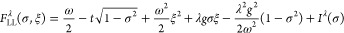
*where the only nonexplicit contribution is*
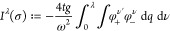


**Figure 7:**
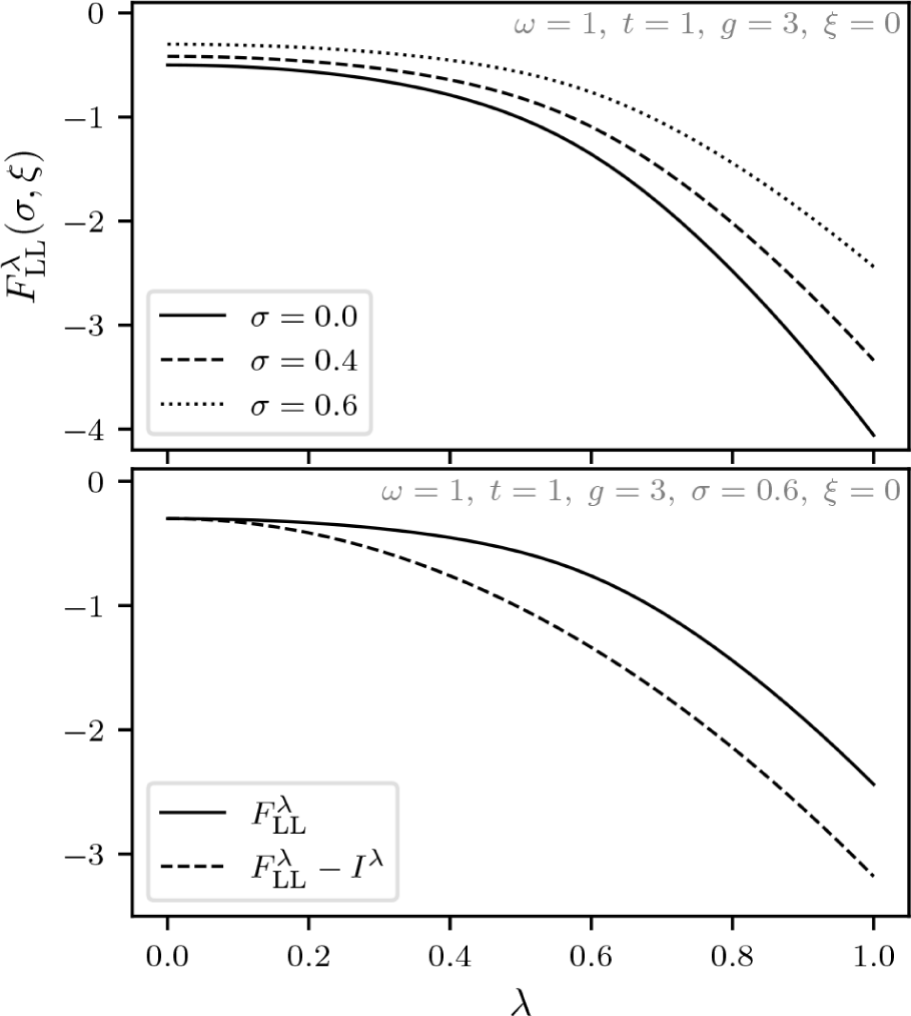
Levy–Lieb functional as a function of λ for ω = 1, *t* = 1, *g* = 3, and ξ = 0. It is obtained numerically as the convex conjugate of the energy functional. The upper panel shows the value of the functional in λ for three values of σ and ξ = 0. The lower panel shows the value of the functional in λ for σ = 0.6 and ξ = 0 (solid line) alongside the explicit terms in the expression for *F*_LL_ in Theorem 5.2 (dashed line), thus depicting the dependence of *F*_LL_ on the nonexplicit correlation energy *I*^λ^.

**Figure 8:**
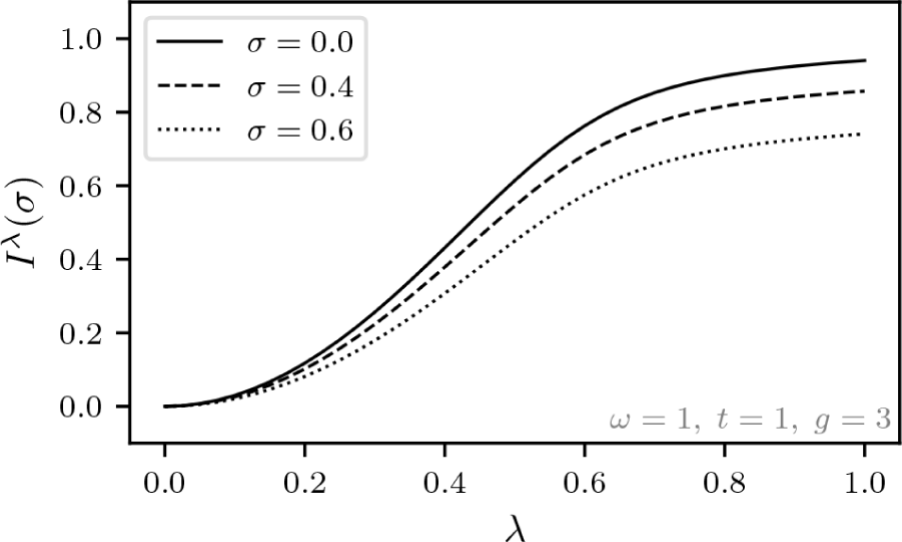
Nonexplicit contribution *I*^λ^(σ) to the adiabatic connection as a function of λ for three values of σ using ω = 1, *t* = 1, and *g* = 3.

In order to visualize the adiabatic connection, we computed the Levy–Lieb functional in λ for different values of σ, see Figure 7 (top panel). Moreover, we compared the full Levy–Lieb functional *F*_LL_^λ^(σ,ξ) to the explicitly known terms by computing *F*_LL_^λ^(σ,ξ) – *I*^λ^(σ), see Figure 7 (bottom panel). This shows, as expected, that the Levy–Lieb functional is concave and decreases with λ. Additionally, as Figure 8 shows more clearly, the nonexplicit term *I*^λ^(σ) is in a positive contribution to the total Levy–Lieb functional that is growing in λ.

The careful reader might wonder why we first employed the displacement rule for *F*_LL_^λ^ and then used the adiabatic connection that connects the zero coupling to any value of λ for *F*_LL_^λ^(σ,0). This is just to obtain the simplest possible expression, since following this route the optimizers are determined at ξ = 0. Using instead the integral representation of *F*_LL_^λ^(σ,ξ) (i.e., never invoking the displacement rule), one instead obtains
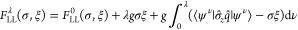
34where ψ^*ν*^ ∈  denotes the optimizer of *F*_LL_^λ^(σ,ξ). Consequently, we have
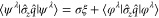
35relating the optimizers at an arbitrary ξ to those at ξ = 0. This relation is also directly seen from the fact noted in Section 4.2 that if ψ is the optimizer of *F*_LL_(σ, ξ) then  is the optimizer of *F*_LL_(σ, ξ + ζ).

Within this basic QEDFT formulation, we thus are able to formulate an almost explicit form (i.e., in terms of the “density” variables only) of the adiabatic connection, where the only nonexplicit term is *I*^λ^(*σ*), which further depends on the optimizers φ^*ν*^ for *ν* ∈ [0, λ]. This is in contrast to the standard DFT setting in which the adiabatic connection remains entirely nonexplicit, see eq 6.

### 5.2 Correlation Contributions

**Figure 9:**
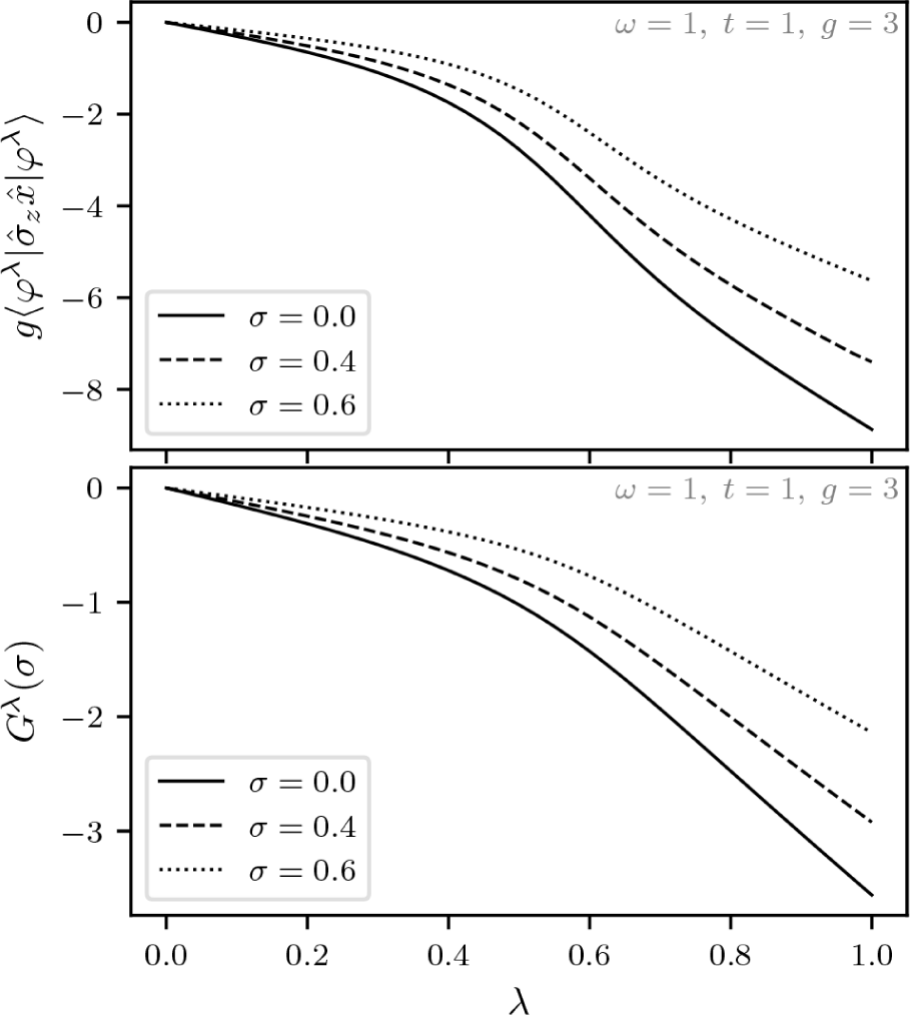
Upper panel shows the expectation value of the coupling, ⟨*g*σ̂_*z*_*q̂*⟩, for the optimizer φ^λ^ at different coupling strengths λ for three choices of σ. The lower panel shows the correlation functional *G*^λ^(σ) as a function of λ, also for three different values of σ. The other parameters are set to ω = 1, *t* = 1, and *g* = 3 for both plots.

To continue the study of *F*_LL_^λ^, we will divide it into different contributions. We begin by noting that the expression given in eq 34 is in full analogy with the adiabatic connection in standard DFT (see eq 6). Motivated by this structure, let us identify the direct-coupling term as
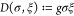
36In analogy to standard DFT we identify the exchange-correlation term
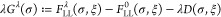
37Naïvely, this term should also depend on ξ, however, using eq 34, we find that for a fixed *g* ∈  and λ > 0,
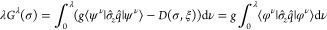
38where the last equality follows from eq 35. It is thus clear that the term *G*^λ^ is independent of ξ. Since this expectation value of the coupling is also the integrand in the adiabatic connection, eq 33, it is interesting to compare this plot to other DFT settings, where an unproven conjecture says that such adiabatic-connection curves must always be convex, see e.g., Crisostomo et al.^72^ (Section 3). While this conjecture was formulated for usual particle interactions, it clearly does not hold in case of the quantum Rabi model as shown in Figure 9 (top panel).

However, if we instead write out eq 37 in terms of the expectation values with respect to φ^λ^ and φ^0^ (using first the displacement rule), we obtain an alternative characterization,
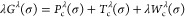
Here, we defined the photonic correlation term,
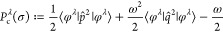
the kinetic correlation term,
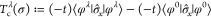
and the correlation from the coupling,
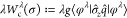
Then, using Theorem 5.2, or alternatively Theorem 4.2.5 and Theorem 4.9, we find that
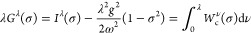
This result corresponds to the perhaps surprising representation of the total exchange-correlation energy in terms of an integral using only the interaction operator in the integrand in standard DFT (while the kinetic correlation energy is still accounted for). We can then express the Levy–Lieb functional at λ as
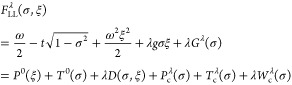
Here, *P*^0^(ξ) = (ω + ω^2^ξ^2^)/2 is the zero-coupling photon energy and *T*^0^(σ) =  is the zero-coupling kinetic energy. The total kinetic functional then is
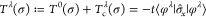
Moreover, recall from Theorem 4.2.4, that
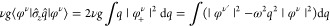
as φ^ν^ is the optimizer of *F*_LL_^ν^(σ,ξ). By insertion into eq 38, we thus obtain the alternative characterization
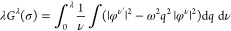


In order to continue the discussion of *G*^λ^(*σ*), recall Perdew’s definition of exchange energy in Section 1.4. Since there is no such thing as a high-density limit in the setting of the quantum Rabi model, we rely on eq 7, where the exchange energy is given as the right derivative of the adiabatic functional at zero coupling minus the direct-coupling term, eq 36,
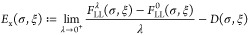
Let the right derivative with respect to λ be denoted by ∂_λ_^+^. Using Theorem 5.2, we have that
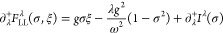
However, by the definition of *I*^λ^(σ),
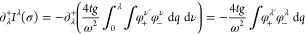
where we recall that φ^λ^ is the optimizer of *F*_LL_^λ^(σ,0). Then, by setting λ = 0, as required for the definition of the exchange energy, we can readily use the optimizer at zero coupling, Theorem 4.9, for ξ = 0. We thus obtain that
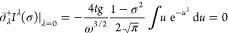
since the integrand is odd. Consequently, all terms of the functional *G*^λ^(*ρ*) are correlation terms, as summarized in the following theorem.

**Theorem 5.3***For every density pair* (σ,ξ) ∈ [−1,1] × *the exchange energy is zero,*
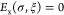
*and the correlation energy is*
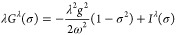


The absence of exchange energy is not a surprising feature for the quantum Rabi model, and neither would be for the Dicke model, since it is two different components, light and matter, that are coupled here and “exchange” only exists between identical, fermionic particles. It is thus clear that the term *G*^λ^(σ) is purely a correlation term. This also motivates our notation λ*G*^λ^ = *P*_c_^λ^ + *T*_c_^λ^ + λ*W*_c_^λ^.

### 5.3 Bounds on Correlation

From Theorem 5.3 we note that the only nonexplicit term in the correlation energy λ*G*^λ^(σ) is *I*^λ^(σ). In order to obtain bounds on the correlation energy, a further analysis of this term is warranted.

To establish a lower bound on the Levy–Lieb functional, suppose that ψ ∈ *Q*_0_ is the ground-state solution of *Ĥ*^λ^(*v*,*j*) with displacement ξ ∈  and polarization σ ∈ (−1,1). Then by eq 18, −*j* = ω^2^ξ + λ*g*σ. Recall from Section 2, in particular the step just before eq 15, that
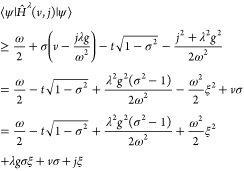
However, since ψ 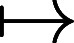 (σ,ξ) then
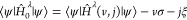
and we obtain the following lower bound for the Levy–Lieb functional
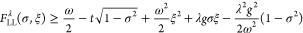
By taking again eq 25 as a trial state for *F*_LL_(σ, ξ), we also obtain the upper bound
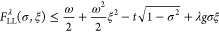
Combining these bounds, we immediately have
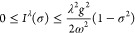
39For the correlation energy this estimate takes the form of a Lieb–Oxford bound.^g^

**Proposition 5.4***The correlation energy satisfies the Lieb–Oxford-type bound*
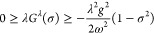


Furthermore, in order to get an alternative upper bound for *F*_LL_^λ^(σ, ξ), suppose another Gaussian trial state
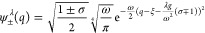
40which satisfies the necessary constraints
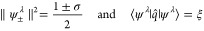
By the displacement rule, Theorem 4.2.2, we may for the sake of simplicity restrict ourselves to the case of ξ = 0. Then by a direct calculation, we obtain that
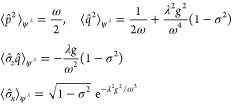
Consequently, we have
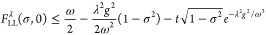
However, this upper bound is very similar to the lower bound given above. In particular, the bounds differ only by the exponential exp(−λ^2^*g*^2^/ω^3^), a factor that rapidly goes to zero as λ increases. Conversely, when λ ≪ ω^3/2^/*g* the exponential is almost one, implying that the upper bound is almost equal to the lower bound. Thus, for λ ≪ ω^3/2^/*g* the trial state, eq 40, is almost the optimizer of *F*_LL_^λ^(σ,ξ).

By use of the new upper bound, an alternative estimate of “kinetic” type follows,
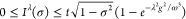
41We can then clearly see that the trial state eq 40 is the exact optimizer in the cases σ = ±1, λ*g* = 0, and *t* = 0 as well as ω → ∞. The first two of these observations are in accordance with the results in Section 4, that the trial state eq 25 is exact in two cases, in the zero coupling case, Theorem 4.9, and for critical polarizations, Corollary 4.6.

### 5.4 Approximate Correlation

**Figure 10:**
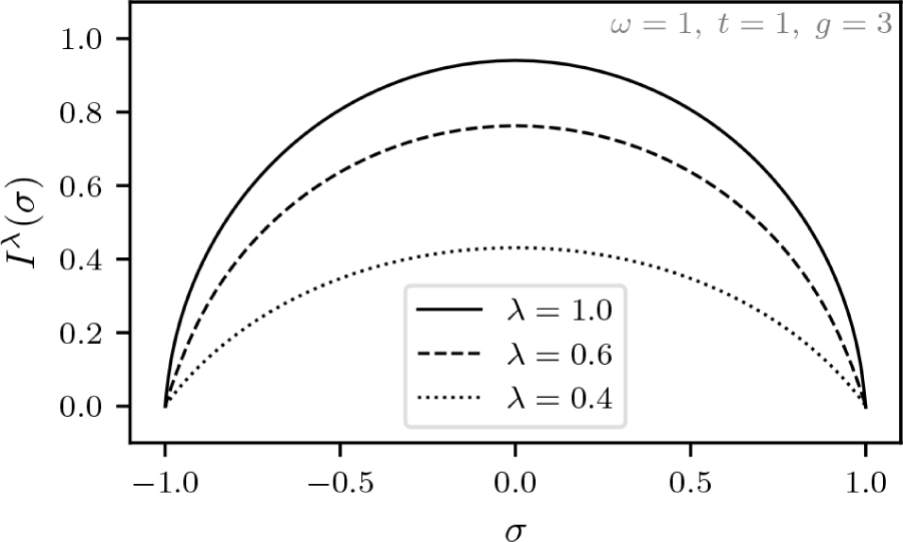
Nonexplicit correlation term *I*^λ^(σ) as a function of σ for three values of λ using ω = 1, *t* = 1, and *g* = 3.

Having established some analytical bounds on the nonexplicit correlation term, let us further investigate the term numerically. In particular, Figure 10 shows the nonexplicit correlation term as a function of the polarization σ for selected values of λ. A numerical investigation for a decreasing sequence of λ shows that *I*^λ^(σ) indeed approaches zero from above as λ → 0, as required by the estimates eqs 39 and 41 and as also suggested by Figure 8. Moreover, we remark that the shape of *I*^λ^(σ) plotted in σ closely resembles the line segment of an ellipse, see Figure 10.

Motivated by this resemblance we make the following ansatz. Suppose three functions (over the parameters λ and *t*) *a*, *b*, and *d* such that
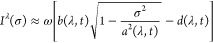
Then by the constraint *I*^λ^(±1) = 0, we have that
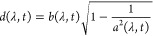
Equipped with this form, let us formulate the following conjecture.

**Conjecture 5.5***For* σ ∈ [−1,1] *the nonexplicit correlation functional is of the approximate form*
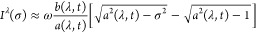
*Here a and b are functions of the parameters λ and t*.

**Figure 11:**
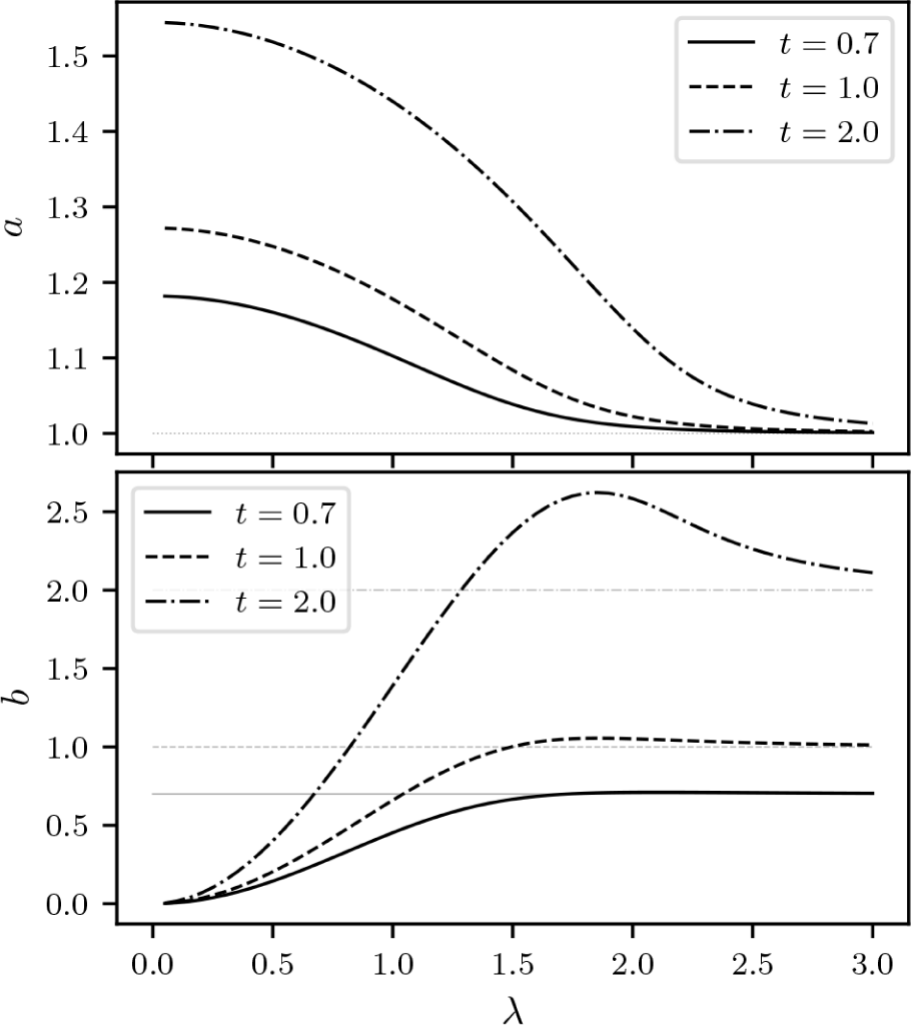
Parameter fitting of the functions *a* and *b* in λ for three select values of *t* at ω = *g* = 1.

**Figure 12:**
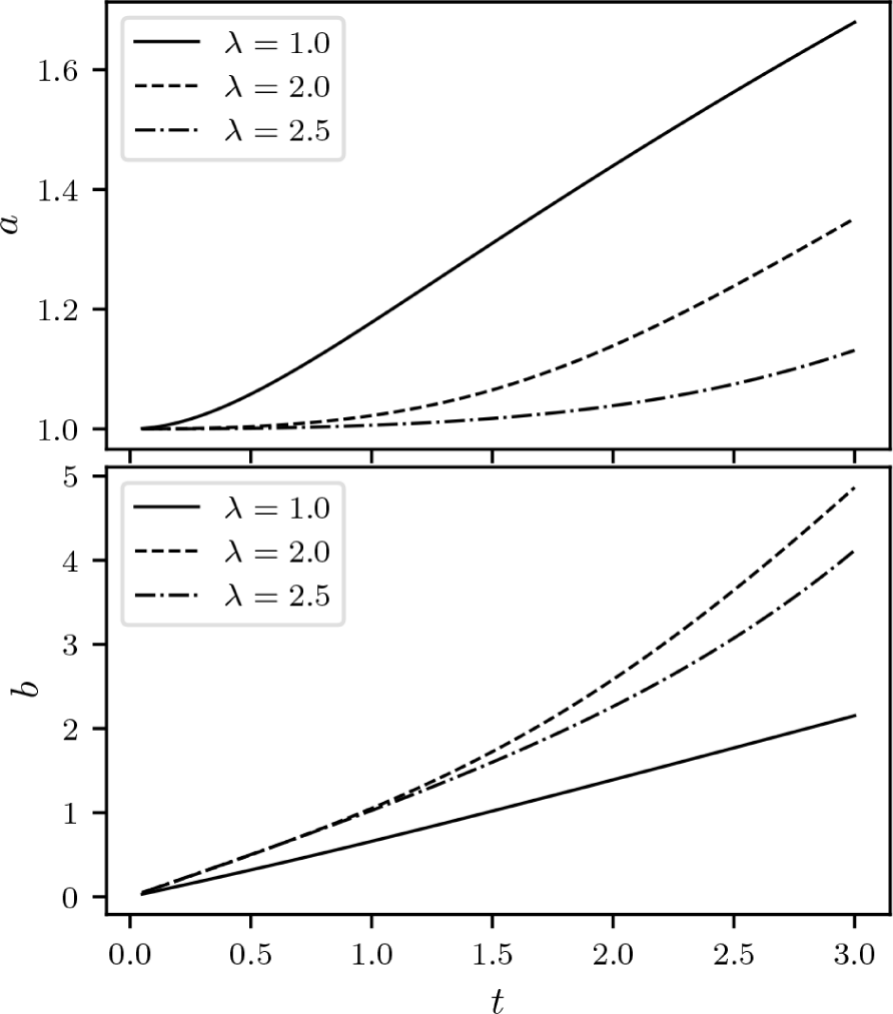
Parameter fitting of the functions *a* and *b* in *t* for three select values of λ at ω = *g* = 1.

To further investigate Conjecture 5.5, let us numerically calculate *I*^λ^(σ) for many combinations of the parameters λ and *t*, for λ, *t* ∈ [0,3] at ω = *g* = 1 . Then by performing a parameter fitting, we obtain *a* and *b* as functions of parameters λ and *t* as shown in Figures 11 and 12. Importantly, the parameter fitting shows that Conjecture 5.5 fits very well with the numerical simulations. In fact, the largest standard deviation in the parameter fitting is only 0.05 (arising in *b*).

From Figures 11 and 12, we learn the following about the functions *a* and *b*.

(1).
(2)
(3) (required by eqs 39 and 41)
(4)
(5)

This shows, in particular, that in the strictly correlated regime (λ = ∞) the nonexplicit part of the correlation functional is
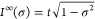
i.e., saturates the upper bound of eq 41. This implies that near the strictly correlated regime, the Levy–Lieb functional is
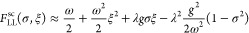
Thus, in the strictly correlated regime, there are no kinetic contributions. This is not unexpected, since the coupling term and the displacement operator are unbounded operators while the kinetic term is bounded by |*t*|.

## 6 Photon-Free Approximation

### 6.1 Effective Potential

In order to derive the *photon-free* approximation, recall the hypervirial relation of eq 21. This relation can be established separately for a noncoupled auxiliary system at λ = 0 (*g* = 0) with ground-state wave function ϕ, which was studied in Section 4.4, and the fully coupled system at λ = 1 with the ground state ψ. In these two cases, eq 21 gives
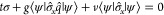
42a
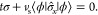
42b

As it is usual in DFT, the potential *v*_s_ for the auxiliary system (which, again in analogy to standard DFT would be called Kohn–Sham system) is chosen such that the value of σ agrees for both systems. This is surely possible if σ ≠ ±1 because of the *v*-representability result from Corollary 4.8.1. In a similar fashion, the values for ξ can be matched between the two systems with a choice of *j*_s_ that follows from the same corollary. However, the choice is also directly visible from the exact hypervirial relation eq 18, which gives
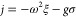
43a
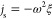
43b

Consequently, the ξ from the coupled system can always exactly be reproduced by choosing the above value for *j*_s_ in the uncoupled system. Now, let us in analogy to standard DFT define the direct-coupling and exchange-correlation potential as *v*_dxc_ = *v*_s_ – *v*. From subtraction of eq 42a we find that
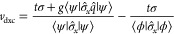
44This means any approximation of the ground-state ψ of the coupled system will approximate the ψ-dependent parts of eq 44. This gives a functional approximation to use for the Kohn–Sham system. The arguably simplest approximation is ψ = ϕ, the mean-field approximation, where ⟨ϕ|σ̂_*x*_*q̂*|ϕ⟩ = ⟨ϕ|σ̂_*x*_|ϕ⟩⟨ϕ|*q̂*|ϕ⟩ = ⟨ϕ|σ̂_*x*_|ϕ⟩ξ since the system is uncoupled and thus matter and photon parts factorize. On the other hand, this approximation misses correlation effects. From eq 44, we then have
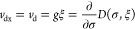
This is just the direct-coupling part, that originates from the coupling term of the Hamiltonian *g*σ̂_*z*_*q̂* ≈ *D*(σ,ξ) = *g*σξ = *v*_d_σ. This means the exchange-only part vanishes, *v*_*x*_ = 0, as was already seen in Theorem 5.3.

**Figure 13:**
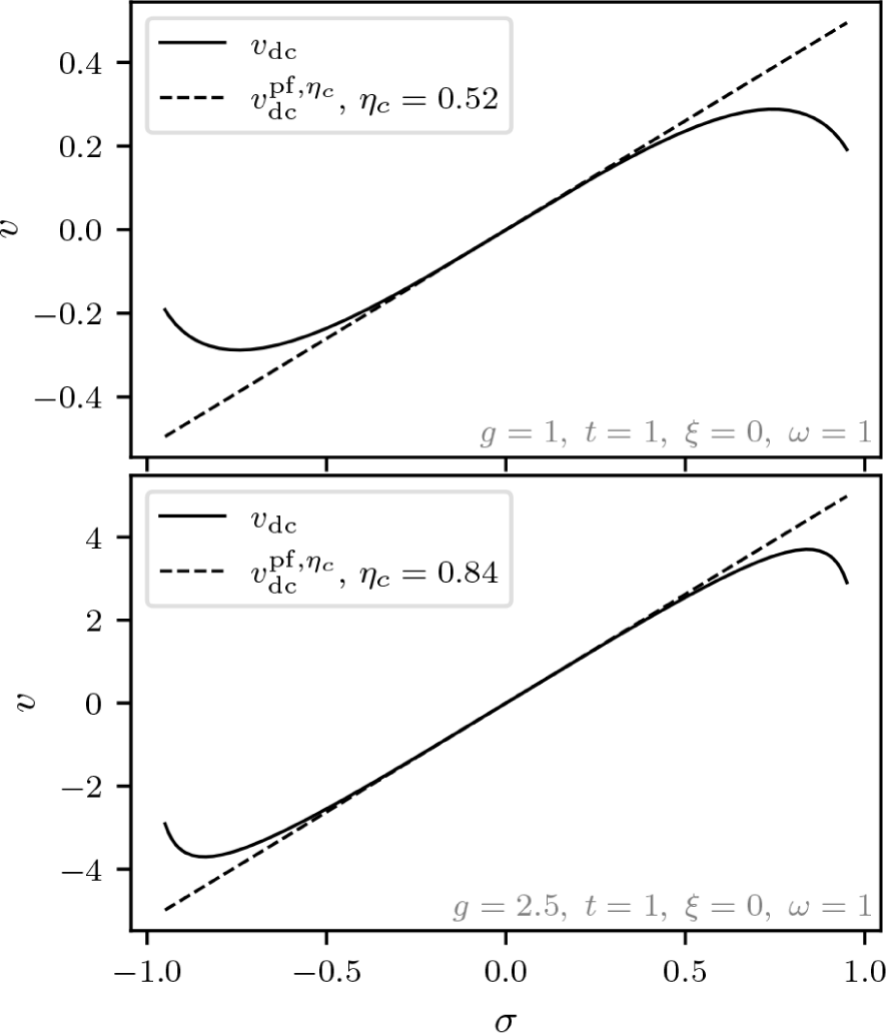
Comparison of the exact direct-coupling and correlation potential *v*_dc_ and the photon-free approximation with correlation factor η_c_, *v*_dc_^pf,η_c_^, plotted as functions of the matter density σ for coupling constants *g* = 1 and *g* = 2.5. The approximation includes the correlation factor η_c_ computed for each *g* from the derivative at σ = 0. Parameters are set to ω = 1, *t* = 1, ξ = 0. The plots demonstrate the effectiveness of the photon-free approximation in reproducing the exact potential, especially not too close to the boundary and at higher coupling strengths.

**Figure 14:**
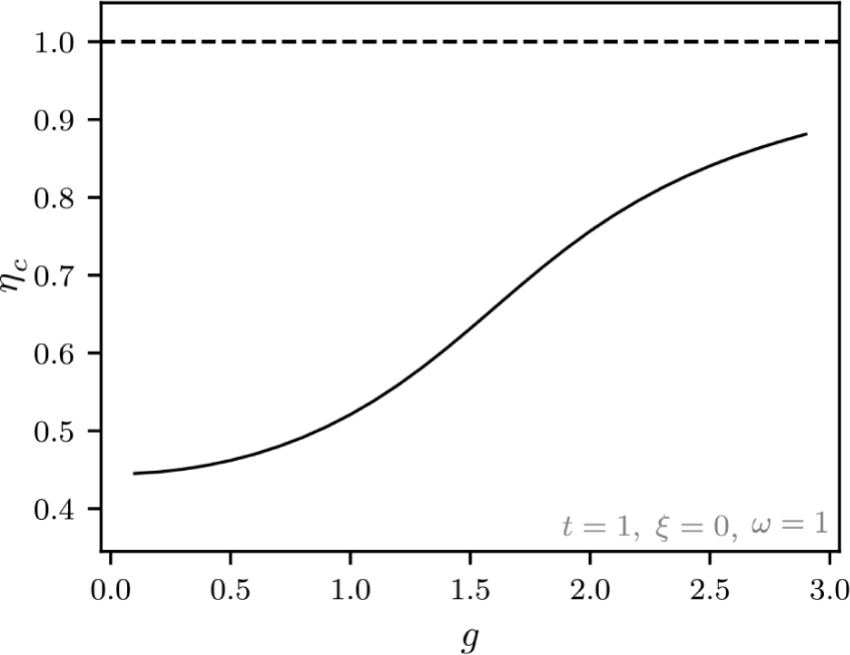
Dependence of the correlation factor η_c_ on the coupling strength *g* for the quantum Rabi model. The plot illustrates how η_c_ approaches 1 as *g* increases, indicating that the photon-free approximation with correlation factor η_c_ becomes exact in the strong-coupling limit for σ not too close to the boundary. The calculations are performed with parameters ω = 1, *t* = 1, ξ = 0. Different numerical cutoffs for maximal photon number are used for various ranges of *g* to ensure numerical accuracy.

We are, however, not limited to this level of approximation and can include some correlation information by using the adiabatic approximation on the level of quantum fluctuations. For a general operator we set *Â* = ⟨*Â*⟩ + Δ*Â*, where Δ*Â* describes the (operator-valued) fluctuations around a mean value that are assumed to be “small”, especially with respect to variation in time (adiabatic approximation). For the displacement operator this means following eq 17 that Δ*q̂* ≈ (−*g*/ω^2^)Δσ̂_*z*_ and consequently that
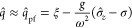
45The same relation can be derived directly from eq 17 by setting d*p̂*/dτ = 0 and eliminating *j* by inserting the exact relation from eq 43a. With the help of eq 45 the photon degree-of-freedom represented by *q̂* can be effectively replaced by matter quantities and we can get an approximate “photon-free” formulation of the problem. This program was already put into effect in QEDFT and forms the basis for one of the first functionals for the Pauli–Fierz Hamiltonian in dipole approximation.^75^ Inserting this photon-free approximation into eq 44 and setting ψ = ϕ yields the functional
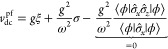
46We realize quickly that the last term vanishes, since σ̂_*x*_σ̂_*z*_ = −*i*σ̂_*y*_ and ⟨σ̂_*y*_⟩ = 0 for any eigenstate by eq 19. This is beneficial since *i*σ̂_*y*_ is skew-adjoint and would thus have imaginary expectation values. We have thus derived an effective potential for a photon-free (uncoupled) system that aims at reproducing the same polarization σ in both systems. A more detailed perturbation-theory analysis including the higher photon states shows that a factor η_c_ < 1 should be introduced to take the matter–photon correlation into account, where η_c_ → 1 in the strong-coupling regime.^34^
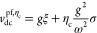
While we will not enter the theoretical details here, a numerical demonstration shows that this leads to a high level of agreement for σ values that are not too close to the boundary and that the linear approximation gets more accurate for strong coupling, see Figure 13. The dependence of the correlation factor η_c_ on *g* is further illustrated in Figure 14.

We can check that this result fully matches the expression for the Levy–Lieb functional along the adiabatic connection from Theorem 5.2. First take the difference between full and zero coupling to get the direct-coupling and correlation energy,
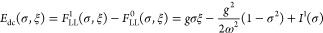
Since the potential corresponds to the negative differential of the respective functional, eq 2, we can directly use differentiation to get the external pair that encodes direct coupling and correlation for *σ* ∈ (−1,1),
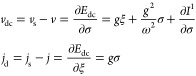
This fits exactly to the previous results. Note that Novokreschenov et al.^45^ recently gave an approximation for the effective potential based on diagrammatic expansion for the quantum Rabi model and the Dicke model.

The effective potential derived in this section aims at reproducing the value of the polarization σ from the fully coupled system in a auxiliary system without light part. We can further ask how well (or if at all) other quantities can be reproduced as well, mainly the ground-state energy or the expectation value of parts of the Hamiltonian. This will be investigated in the next section by defining a whole photon-free Hamiltonian instead of just an effective potential.

### 6.2 Photon-Free Hamiltonian

**Figure 15:**
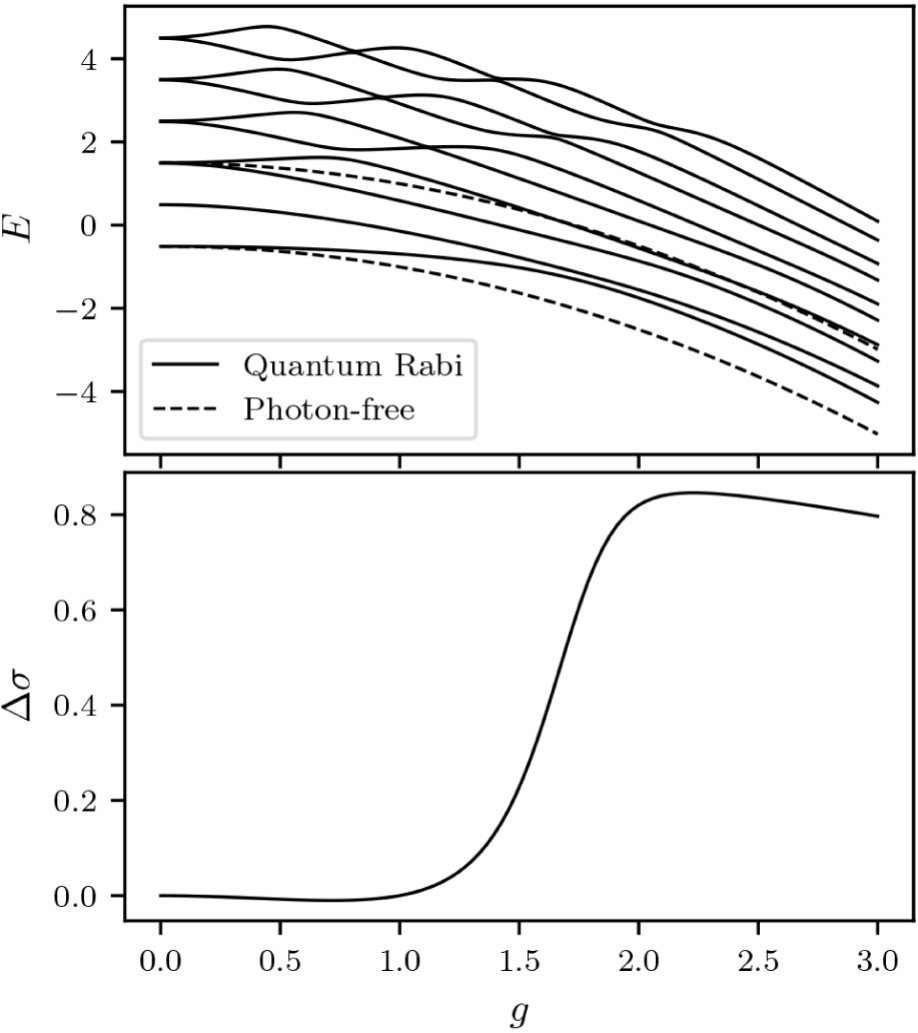
Upper plot shows the spectrum of the quantum Rabi Hamiltonian *Ĥ*(*v*,*j*) with ω = 1, *t* =1, *v* = 0.1, *j* = 0.1 at different couplings *g* together with the spectrum of its photon-free approximation *Ĥ*_pf_(*v*,*j*) (dashed lines). The lower panel compares the expectation values of σ̂_*z*_ between the ground state ψ of *Ĥ*(*v*,*j*) and ψ_pf_ of *Ĥ*_pf_(*v*,*j*), Δσ = ⟨ψ|σ̂_*z*_|ψ⟩ – ⟨ψ_pf_|σ̂_*z*_|ψ_pf_⟩.

The previous, adiabatic approximation for operators allowed us to replace the photonic degree-of-freedom *q̂* by pure matter quantities in eq 45 and derive a correlation approximation. But we can go one step further and not only substitute this potential into the uncoupled Hamiltonian, but also provide a photon-free expression for the field energy. For this, remember that the ladder operator is
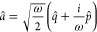
We take the photon-free approximation for *q̂* from eq 45. For the momentum operator we simply choose *p̂*_pf_ since we have *p̂* = d*q̂*/dτ ≈ 0 from eq 17 and the adiabatic approximation. This means we get
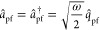
which is now a self-adjoint operator (since there are no more photons to create or annihilate). The photon energy is thus approximated by
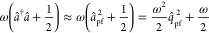
By insertion of eq 45 for *q̂*_pf_ and using ξ = −(*g*σ + *j*)/ω^2^ from eq 43a, we get a photon-free version of the quantum Rabi Hamiltonian of eq 9,
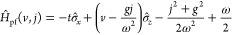
We notice that the only difference to the Hamiltonian of the two-level system alone are the shift −*gj*/ω^2^ in the potential *v*, exactly what we derived before as *v*_dc_^pf^ in eq 46 if we set *j* = −ω^2^ξ – *g*σ again, and an overall shift in the energy. The spectra of the Hamiltonians and the difference in the expectation values of σ̂_*z*_ in the ground state are compared in Figure 15. Note that while the ground-state energy agrees only for small coupling, the expectation values of σ̂_*z*_ will also match again in the strong-coupling limit, where *v*_dc_^pf^ gives the exact direct-coupling and correlation potential as discussed in Section 6.1.

Finally, we point out that the photon-free approximation contains an entirely new ingredient when applied to the Dicke model. There, the effective potential at one site will include information about the polarization at other sites and thus amounts to an effective interaction between the two-level systems.

## 7 Conclusions

In summary, this work presents a thorough convex-analytical treatment of ground-state DFT applied to the most basic QED models, in particular the quantum Rabi model and the Dicke model. In the quantum Rabi model, not only can we achieve full *v*-representability for all polarizations (except σ = ±1) and displacements, but we are also able to derive quite explicit expressions for the adiabatic connection and the photon-free approximation. While many of the discussed features for a QEDFT will generalize from the minimal setting of the quantum Rabi model to more complex settings like the (multimode) Dicke model,^22^ we do not have all results assured. Especially, it is an open question if the properties of the ground state from Section 2.3 still hold for the Dicke model, and, as a derived property, if *v*-representability holds for the Dicke model.

With the quantum Rabi and Dicke models on solid grounds, the developed methodologies to analyze the properties of ground states and the corresponding QEDFT formulations can be extended to more challenging setups. While ultimately a characterization of the full (continuous) Pauli–Fierz problem would be desirable, already small modifications of the Dicke model might change the physics substantially. This is specifically so if we reinterpret the Dicke Hamiltonian in the length-gauge.^39,40^ The length gauge is a unitary transformation of the Pauli–Fierz Hamiltonian in Coulomb gauge under the long-wavelength approximation, and mixes light and matter degrees of freedom in its basic coordinates.^9^ In this case, the Dicke Hamiltonian gets modified and already includes direct dipole–dipole interactions between the different two-level systems. This type of interaction is distinct from the usual Coulomb interaction and is currently assumed to be responsible for many of the observed effects in polaritonic chemistry for macroscopic ensembles of molecules under vibrational strong coupling.^41,76^ Investigating this setting for the zero-temperature case as well as in thermal equilibrium is an obvious and very timely next step. Here, an important physical difference between the usual electronic DFT and many problems addressed with QEDFT becomes apparent: Due to a photonic structure that enhances certain photonic modes, different length-scales begin to talk to each other and we can no longer consider different molecules as statistically independent, not even in the dilute-gas limit. Thus, QEDFT methodologies become important even for the case of simplified matter subsystems, such as just two-level systems, because of the large number of coupled matter systems in macroscopic ensembles.

## Data Availability

The source code for performing the numerical investigation is available on GitHub: https://github.com/VegardFalmaar/QEDFT-Quantum-Rabi-Code.

## Footnotes

a This way, the set of *v*-representable densities depends on the selected set of allowed potentials. A natural choice is *L*^3/2^ + *L*^∞^ as explained later in this section.

b Instead of the standard convex conjugate *f**(*y*) = sup_*x*_(⟨*y*, *x*⟩ – *f*(*x*)), we use *E*(*v*) = *F*^∧^(*v*) = −*F**(−*v*) and *F*(ρ) = *E*^∨^(ρ) = (−*E*)*(−ρ).

c That is units such that ℏ = *m*_*e*_ = *e* = 4πε_0_ = 1.

d In this case we identify the parity transformation by its usual action of reflection through the origin, i.e., : *q* −*q*. The time conjugation, is identified by its usual action of interchanging polarization/spin, i.e., : σ  −σ, even though the theory presented here is time-independent.

e The argument of Lieb in standard DFT has also analogous counterparts in paramagnetic CDFT.^69,70^

f Note that this relation is true in general for a conjugate pair. However, for simplicity, in this work we only state this for λ = 0 where all expressions are explicitly known.

g We remind the reader that the Lieb–Oxford bound^73^ (of standard DFT) states that the indirect Coulomb energy of a normalized wave function ψ is bounded below by , *C* > 0. The bound presented here is similar in spirit to the one-dimensional version.^74^
